# Augmenting Critical Care Patient Monitoring Using Wearable Technology: Review of Usability and Human Factors

**DOI:** 10.2196/16491

**Published:** 2021-05-25

**Authors:** Evismar Andrade, Leo Quinlan, Richard Harte, Dara Byrne, Enda Fallon, Martina Kelly, Siobhan Casey, Frank Kirrane, Paul O'Connor, Denis O'Hora, Michael Scully, John Laffey, Patrick Pladys, Alain Beuchée, Gearoid ÓLaighin

**Affiliations:** 1 Electrical & Electronic Engineering, School of Engineering National University of Ireland, Galway Galway Ireland; 2 Human Movement Laboratory CÚRAM Centre for Research in Medical Devices National University of Ireland, Galway Galway Ireland; 3 Physiology, School of Medicine National University of Ireland, Galway Galway Ireland; 4 General Practice, School of Medicine NUI Galway Galway Ireland; 5 Irish Centre for Applied Patient Safety and Simulation (ICAPSS) University Hospital Galway Galway Ireland; 6 Mechanical Engineering, School of Engineering NUI Galway Galway Ireland; 7 Intensive Care Unit University Hospital Galway Galway Ireland; 8 Medical Physics and Clinical Engineering, University Hospital Galway Galway Ireland; 9 School of Psychology NUI Galway Galway Ireland; 10 Anaesthesia, School of Medicine NUI Galway Galway Ireland; 11 Department of Anaesthesia & Intensive Care Medicine Galway Ireland; 12 Centre Hospitalier Universitaire de Rennes (CHU Rennes) Rennes France; 13 Faculté de Médicine de l’Université de Rennes Rennes France

**Keywords:** patient monitor, physiologic monitor, human factors, ergonomics, usability, user experience, wearable, mobile phone, critical care

## Abstract

**Background:**

Continuous monitoring of the vital signs of critical care patients is an essential component of critical care medicine. For this task, clinicians use a patient monitor (PM), which conveys patient vital sign data through a screen and an auditory alarm system. Some limitations with PMs have been identified in the literature, such as the need for visual contact with the PM screen, which could result in reduced focus on the patient in specific scenarios, and the amount of noise generated by the PM alarm system. With the advancement of material science and electronic technology, wearable devices have emerged as a potential solution for these problems. This review presents the findings of several studies that focused on the usability and human factors of wearable devices designed for use in critical care patient monitoring.

**Objective:**

The aim of this study is to review the current state of the art in wearable devices intended for use by clinicians to monitor vital signs of critical care patients in hospital settings, with a focus on the usability and human factors of the devices.

**Methods:**

A comprehensive literature search of relevant databases was conducted, and 20 studies were identified and critically reviewed by the authors.

**Results:**

We identified 3 types of wearable devices: tactile, head-mounted, and smartwatch displays. In most cases, these devices were intended for use by anesthesiologists, but nurses and surgeons were also identified as potentially important users of wearable technology in critical care medicine. Although the studies investigating tactile displays revealed their potential to improve clinical monitoring, usability problems related to comfort need to be overcome before they can be considered suitable for use in clinical practice. Only a few studies investigated the usability and human factors of tactile displays by conducting user testing involving critical care professionals. The studies of head-mounted displays (HMDs) revealed that these devices could be useful in critical care medicine, particularly from an ergonomics point of view. By reducing the amount of time the user spends averting their gaze from the patient to a separate screen, HMDs enable clinicians to improve their patient focus and reduce the potential of repetitive strain injury.

**Conclusions:**

Researchers and designers of new wearable devices for use in critical care medicine should strive to achieve not only enhanced performance but also enhanced user experience for their users, especially in terms of comfort and ease of use. These aspects of wearable displays must be extensively tested with the intended end users in a setting that properly reflects the intended context of use before their adoption can be considered in clinical settings.

## Introduction

### Challenges in Critical Care Patient Monitoring

**Figure 1:**
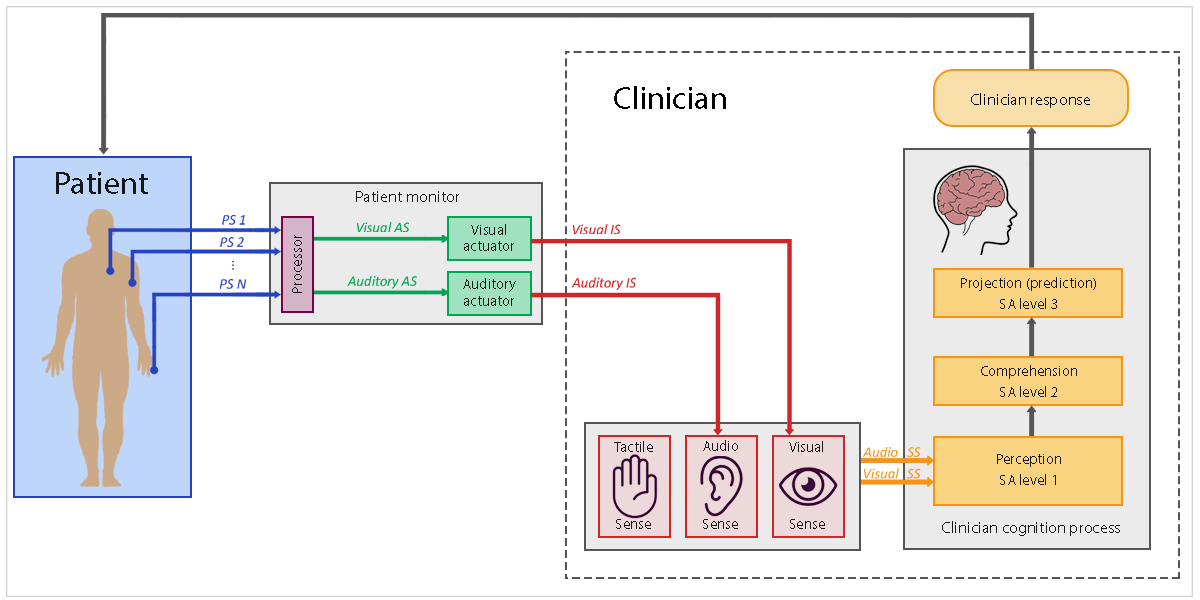
Patient monitor interaction mechanisms with the clinician. The patient’s physiological state is conveyed to the clinician through their visual and auditory senses. Once the clinician perceives a change in the patient’s state through these sensory signals, their cognition processes make use of this information (in addition to other contextual information) to comprehend the patient’s current state and make projections of their future state. At the end of this process, clinicians can make a decision on what they should do next regarding the patient’s care. AS: actuator signal; EDS: external device signal; IS: interaction signal; PS: physiological signal; SS: sensory signal.

Monitoring the vital signs of patients is a crucial task when dealing with critical care patients [1,2]. For this task, critical care clinicians extensively use a patient monitor (PM), which is typically placed close to the patient in the intensive care unit (ICU) or operating room. The essential features of a PM used for critical care patient monitoring were presented by Andrade et al [3]. The PM uses sensors connected to the patient to measure a range of *physiological signals* (eg, heart rate [HR], blood pressure [BP], and saturation of peripheral oxygen [SpO_2_]). This information is processed, converted into a human-readable format (eg, digital values and traces), and presented to the clinician through the PM screen. In addition, when the PM detects any sign of abnormality in the patient’s vital signs (eg, elevated HR), it alerts the user of the potential risk to the patient through the auditory alarm system. These interaction mechanisms between the PM and the clinician are presented in Figure 1.

These interaction processes enable the clinician to be continually informed about the patient’s state. As discussed by Andrade et al [3], the PM is used in a variety of critical care settings (eg, ICUs, high dependency units, and operating theaters). Each of these different settings puts different demands on the PM, and although this device is designed as a generic patient monitoring device, some challenges are associated with using the PM to monitor critical care patients in some specific contexts of use. For example, during an anesthesia procedure, anesthesiologists need to check the patient’s skin pallor, chest movement, and other signs, while also continuously being required to check the PM for the patient’s vital signs [4,5]. In this case, the clinician’s *visual sense* is required for several tasks simultaneously, which increases the likelihood of the clinician missing a critical event. This can be even more problematic when, because of limited space, the PM is not in the anesthesiologist’s direct line of vision [6]. This ergonomic issue not only impacts the anesthesiologist’s physical comfort but can also lead to human error [7].

Another well-documented context-of-use challenge for a PM is the noise generated by PM alarms and the associated alarm fatigue [8,9]. ICU nurses, for example, may be exposed to as many as 700 alarms (from multiple alarming medical devices) per patient per day [10,11]. In addition, depending on the ICU layout, multiple patients might be monitored in the same area, which increases the number of alarms significantly. As the ICU nurse must be notified immediately if the vital signs become abnormal, they must be close enough to the PM to be able to hear an alarm. This cacophony of alarms may disturb their workflow and distract them, especially in situations where they are already under stress or involved in other essential activities related to the patient’s care [12].

In an attempt to improve patient monitoring in critical care, several researchers have developed novel interface designs to augment the PM [3]. In other studies, researchers have attempted to minimize the problem of alarm fatigue with various techniques such as developing better signal filtering algorithms, changing the PM settings, and changing hospital protocols (eg, frequently changing electrocardiogram electrodes, which might otherwise lose contact because of poor adhesiveness) [13]. With the advancement of wearable technology in a range of application areas, researchers have sought to investigate how wearable devices may be used to enhance patient monitoring by overcoming these identified problems and thus potentially improve the experience of the clinicians and, therefore, potentially enhance their performance. Our review focuses on the use of wearable devices to address the identified problems associated with the PM in critical care medicine.

### Augmenting Patient Monitoring With Tactile Displays

**Figure 2:**
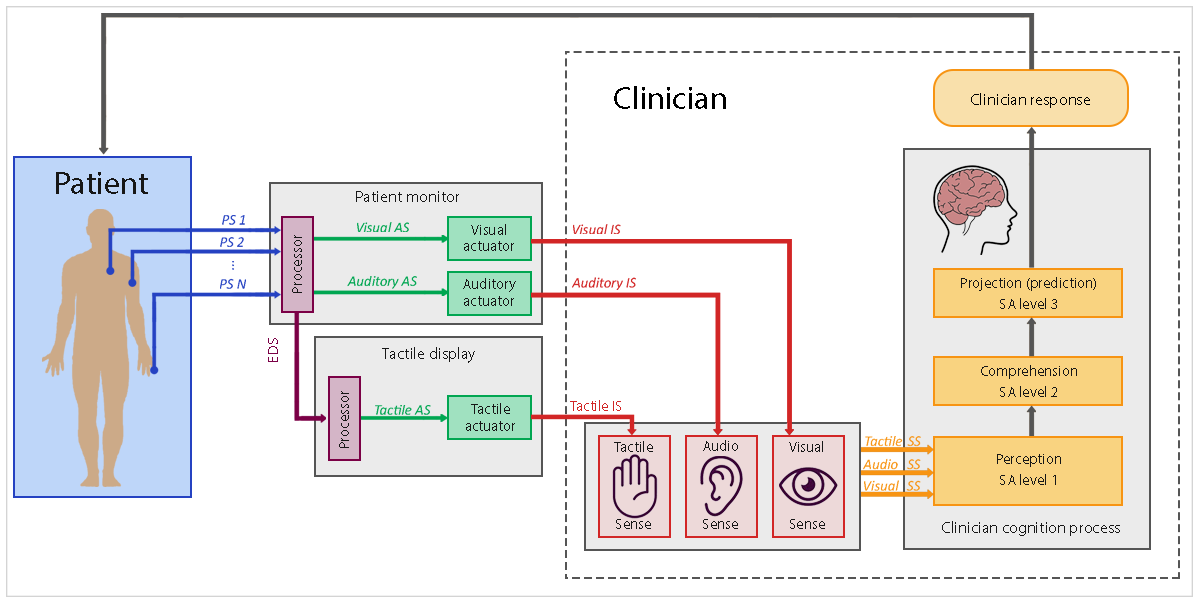
When a tactile display augments a patient monitor, the tactile display receives the patient data from the patient monitor, and this information is conveyed to the clinician using the clinician’s tactile sense through the delivery of vibration sequences. Tactile displays can be attached to different parts of the clinician’s body, such as the wrist, forearm, and waist. AS: actuator signal; EDS: external device signal; IS: interaction signal; PS: physiological signal; SS: sensory signal.

As illustrated in Figure 1, the PM conveys patient information to clinicians visually and aurally. Tactile displays, on the other hand, are composed of small devices (tactors) that use vibratory sequences to display the patient status to the clinician. Therefore, the goal of tactile displays is to enhance the patient monitoring task by using the clinician’s *tactile sense* in addition to their *visual* and *audio* senses, which are already being used by the PM (Figure 2).

In addition to information coding using the vibration time sequences, designers may also use the intensity of the vibration and the position of the tactors as means to display additional information. For example, the intensity of the vibration can be used to convey the extent of a change in a variable value with a low amplitude change encoded as a low-intensity vibration and a high amplitude change encoded as a high-intensity vibration. The location of the tactors can be used to represent the relative value of a variable (eg, tactors vertically positioned in the arm can be programmed to indicate an increase or decrease in the variable value by activating the tactors in sequence upwards or downwards) and to represent a specific physiological measure (eg, a tactor on the left arm representing SpO_2_ and a tactor on the right arm representing HR) [14]. Therefore, designers can use a series of combinations and permutations with tactile parameters to display patient information.

The tactile display uses a *processed* version of the data presented by the PM screen. For example, it might display whether a particular physiological signal is increasing, decreasing, or not changing (*continuous display*), or it can be used to display alarms in a modified way to that delivered aurally by the PM (*alarm display*). Continuous tactile alarm displays could be used to support the anesthesiologist during anesthesia procedures by informing the anesthesiologist of the patient’s state without having to avert their eyes from the patient multiple times during a procedure. When configured as an alarm display, the vibration pattern delivered by the tactor may indicate a PM alarm status (eg, low risk, high risk, or technical alarm), and the body site of the vibration could indicate which parameter is the subject of the alarm. The anesthesiologist could use this tactile display configuration to be informed only when a variable value becomes abnormal, without having to look at the PM screen to establish which variable is generating the alarm. ICU nurses could also use alarm tactile displays to reduce the number of audio alarms in the ICU. For example, instead of the PM sounding an alarm to everyone in the ICU, alarms would be silently directed to the nurse looking after that particular patient, using a tactile display.

### Augmenting Patient Monitoring With Head-Mounted Displays

Another approach to solving the problem of anesthesiologists having to divert their visual attention from the patient to the PM screen is the use of head-mounted displays (HMDs). The patient’s vital signs can be displayed directly on the HMD, allowing the anesthesiologist to observe the vital signs regardless of where their gaze is directed. Designers have the option to display the same information presented by the PM screen or provide a subset of that information (eg, only the digital values).

**Figure 3:**
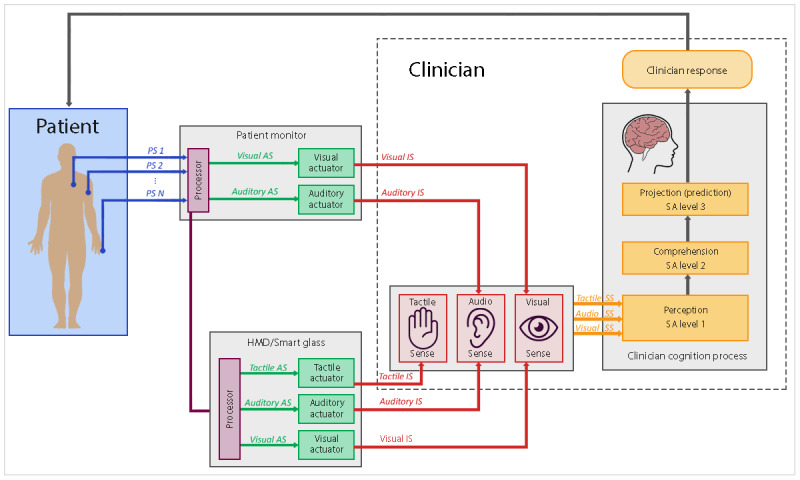
Interaction mechanism between the head-mounted display and the clinician. AS: actuator signal; EDS: external device signal; IS: interaction signal; PS: physiological signal; SS: sensory signal.

The initial HMDs were bulky prototypes with a wired connection to a computer. However, in 2013, the first smart glass was launched, Google Glass (Google LLC). This device is an optical HMD in the form factor of a pair of eyeglasses. When used for vital sign monitoring, Google Glass has the potential benefit of improved comfort because of its size (13.3 cm×20.3 cm), mass (36 g), and wireless design. The display is positioned on the right side of the right eye. HMDs and smart glasses may also be used to monitor patient alarms from multiple patients in an ICU. For example, ICU nurses could wear smart glasses to display when the vital signs of one of his or her patients become abnormal. As can be seen in Figure 3, in addition to their inherent visual actuator, HMD or smart glasses can also feature tactile and auditory actuators. Audio can be transmitted to the user through bone conduction, and vibration sequences can be conveyed by placing a small tactor on the device. Therefore, designers have the option to combine these 2 additional interactive elements to enhance interaction with the clinician.

### Augmenting Patient Monitoring With Smartwatches

Another wearable being explored by researchers for patient monitoring is the smartwatch, connected to the wireless network either directly or through the user’s smartphone or tablet. Most apps developed for smartwatches for health care monitoring focus on its use as a sensor to monitor the wearer’s vital signs or health status [15]. However, given the increasing power of smartwatches, researchers are starting to investigate the feasibility of clinicians wearing smartwatches for patient vital sign or alarm display applications in critical care settings.

**Figure 4:**
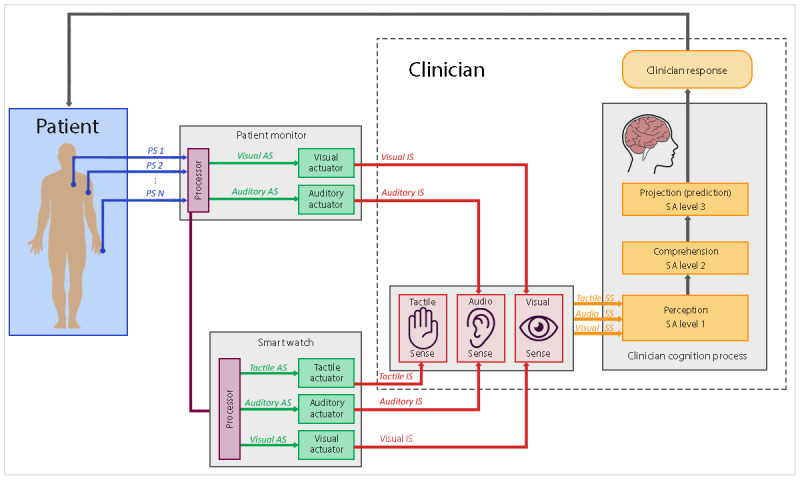
Interaction mechanism between the smartphone/smartwatch and the clinician. AS: actuator signal; EDS: external device signal; IS: interaction signal; PS: physiological signal; SS: sensory signal.

As shown in Figure 4, smartwatches can use 3 senses to convey information to the clinician.

Given the described challenges of monitoring critical care patients using PMs and the opportunities for wearable devices to address these challenges, the authors found it timely to investigate the state of the art in wearable devices applied to critical care patient monitoring. This study aims to critically review the literature on wearable devices in critical care medicine in terms of design, performance, and usability and to explore how the participants in the different studies responded to the use of these wearable devices. This review critically analyzes the relevant literature, with a focus on the usability and human factors performance of the prototype devices reviewed.

## Methods

### Article Selection

A narrative synthesis approach was used in this scoping review. Although this is not a systematic review, the papers selected for review were identified using PRISMA (Preferred Reporting Items for Systematic Reviews and Meta-analyses) [16]. The search by article title, abstract, and keywords was conducted in 4 relevant databases (Scopus, PubMed, Cochrane Library, and Engineering Village) using the keywords presented in Textbox 1.

**Keywords used in the database search. The keywords are grouped into 4 categories: keywords related to wearable devices, usability and human factors, hospital settings, and vital sign monitoring.**

Wearable devices (AND)

- “wearable” OR “tactile” OR “head-mounted” OR “google glass” OR “smart glasses” OR “smartwatch” OR “smart watch”

Usability and human factors (AND)

- “human factor*” OR “usability” OR “ergonomic*” OR “human error” OR “UX” OR “user experience” OR “situation* awareness” OR “response time” OR “detection time” OR “performance” OR “accuracy” OR “efficiency” OR “effectiveness” OR “satisfaction”

Hospital settings (AND)

- “hospital” OR “intensive care” OR “ICU” OR “critical care” OR “operating room” OR “emergency department” OR “cardiology” OR “surgery” OR “an*sthesia”

Vital signs monitoring

- “vital sign” OR “heart rate” OR “spo2” OR “blood pressure” OR “respiratory rate” OR “h*modynamic” OR “alarm” OR “monitoring parameter” OR “physiologic*”

The literature search included data up to May 2020, with no cutoff on the start date. Articles were further excluded after title, abstract, and full paper analysis by members of the multidisciplinary team (composed of engineers, health scientists, nurses, anesthesiologists, human factors specialists, and medical consultants). To ensure that all the relevant studies were identified, the team reviewed each paper’s references, looking for possible studies that were not captured with our search strategy, and 1 study was identified [3].

### Inclusion and Exclusion Criteria

The focus of the review is on the human factors and usability of prototype wearable devices from research laboratories designed to augment PMs to enhance patient monitoring and to overcome PMs’ identified limitations in critical care medicine. On the basis of this focus, the inclusion criteria used in this review were as follows:

1. Studies must be published in English and appear in peer-reviewed academic sources.
2. The prototype display must be a wearable device designed for real-time physiological monitoring or feedback in critical care.
3. The study must include user testing of the prototype display and present the test findings.

### Data Analysis

The data analysis involved carefully reviewing each paper to extract the following information and present it in a summarized form in the paper:

1. Display modality: for example, tactile, auditory, and visual
2. Intended user: for example, nurse, surgeon, and anesthesiologist
3. Intended use:
  Single or multiple patient monitoring
  Continuous vital sign monitoring or alarm condition alert
4. Study design adopted to evaluate the display:
  The participant’s clinical expertise
  The environment in which the device was evaluated
  Simulated or real clinical procedure used
  Control device adopted
  Outcome measures used
  Usability and clinical performance evaluated
  Within-subject or between-subject design

## Results

### Overview

**Figure 5:**
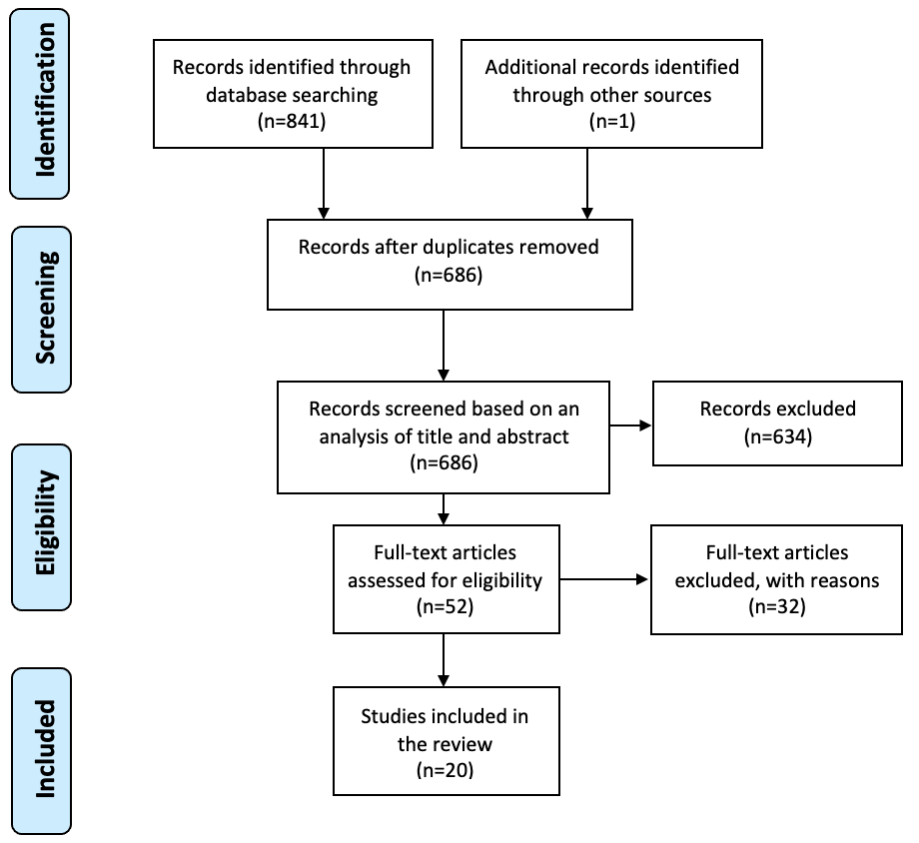
PRISMA (Preferred Reporting Items for Systematic Reviews and Meta-analyses) guidelines flow diagram depicting how many records were identified, screened, assessed, and included in the review.

A breakdown of the article search using the PRISMA guidelines can be seen in Figure 5.

In the *identification* phase of the review, the search of the databases, using the chosen keywords described in Textbox 1, provided a total of 841 records. In the *screening* phase, duplicate records were removed, resulting in 684 remaining records. These were reviewed by title and abstract. We identified that 634 studies clearly did not meet the inclusion criteria and were therefore discarded. In the *eligibility* phase, the full text of the remaining 52 studies was examined in more detail*,* and a further 32 studies were excluded for not meeting the inclusion criteria. The 20 remaining studies were *included* in this review. In reporting on these studies, a standardized method of reporting on the terminology and performance variables was created, as different studies used different names for the same parameters and other names for the same technology or techniques, which could create confusion for the reader. Therefore, a mapping between the new standardized naming convention and the other names was created and is presented in Multimedia Appendix 1. The studies included were grouped into 3 categories, depending on the type of wearable device involved. A total of 10 studies investigated the use of *tactile displays*, 10 studies investigated the use of *HMD* or smart glasses, and 1 study investigated the use of *smartwatches.*

### Tactile Displays

**Figure 6:**
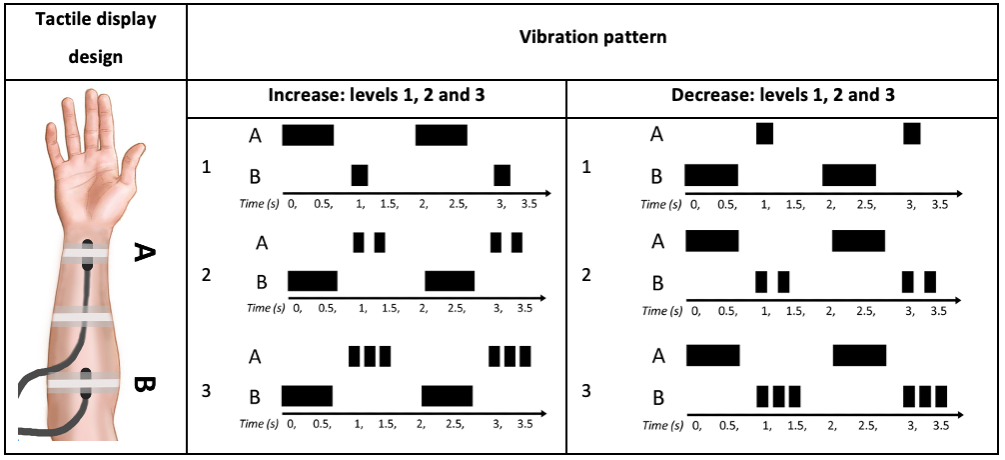
Tactile display on the forearm containing 2 tactors (A and B). The prototype was intended to monitor a single variable with 6 distinct vibration patterns: 3 to represent different levels of increase and 3 to represent different levels of decrease in the monitored variable. The black-block pattern indicates the tactile at that location is activated at that point in time. Note that the increase and decrease patterns are the same except that the A and B sites are interchanged (a model of the concept presented in the paper).

A total of 10 studies investigated the use of tactile displays as patient monitoring devices for critical care. The first investigation of tactile displays for anesthesia monitoring was conducted by Ng et al [17]. Ng et al [17] developed a tactile display prototype composed of 2 vibrating motors located on the forearm (Figure 6). These vibration motors generated 6 different alarms, provided by 6 different vibration patterns, corresponding to a +10%, +20%, +30%, −10%, −20%, and −30% change in the variable of interest. The tactile display was compared with an auditory display, which provided 6 different alarms, provided by 6 different auditory patterns, depending on the variable change level and direction.

**Figure 7:**
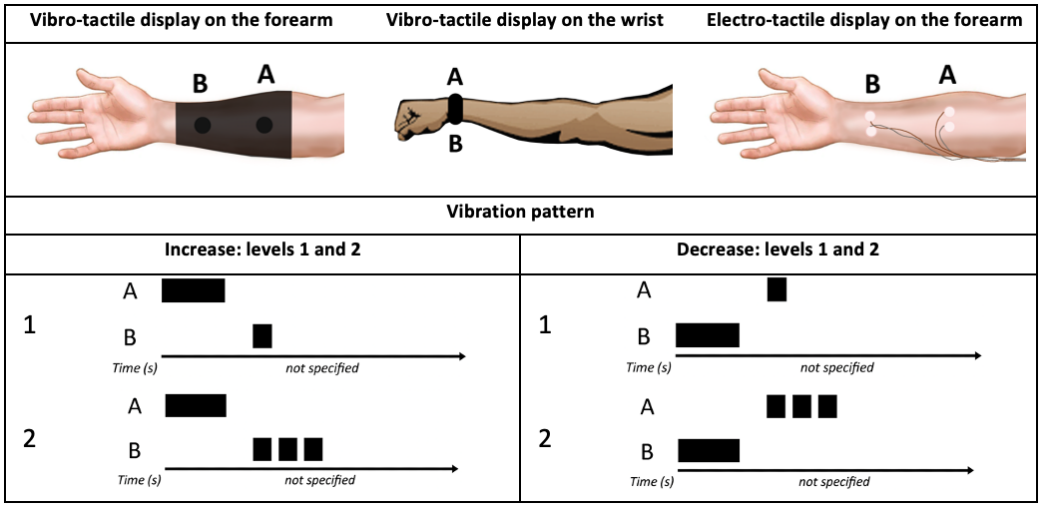
Three tactile displays monitoring a single variable using the same vibration/electrical stimulation pattern. The electro-tactile display on the forearm stimulated mechanoreceptors at 2 forearm locations (a model of the concept presented in the paper).

A total of 10 engineering students with no anesthesia training were asked to test the tactile display, an auditory display, and a combination of these 2 displays. The interaction signal (IS) *detection* by the participants was statistically significantly better when using the tactile display or a combination of the tactile display and auditory display than when using the auditory display alone. Six participants commented on the auditory display’s poor ability to attract attention, which explains the faster IS *detection* for the tactile display. On the other hand, regarding usability, 9 participants reported some discomfort with the wearables, citing arm numbness, resulting from the tightness of the elastic strips; itchiness caused by the vinyl sheet connecting the vibrating motors; and a restriction of arm motion from the nonwireless tactile prototype. Two years later, Ng et al [18] evaluated a new vibrotactile display on the forearm, a vibrotactile display on the wrist, and an electro-tactile display on the forearm. The vibrotactile display on the forearm and the vibrotactile display on the wrist used direct current motors to generate vibrations at the forearm (tactors), and the electro-tactile display on the forearm used a low voltage (9 V) nerve stimulator in the forearm skin to convey information (Figure 7). The study aimed to identify which mechanism was more suitable for a tactile display (electro-tactile or vibrotactile) and the preferred location on the body for it to be located (wrist or forearm). It was found that the mechanical vibration was superior to the electrical stimulation in terms of *learnability* and *IS identification*. Participants (26 individuals with no medical training) experienced discomfort when using the electro-tactile display prototype and found it more challenging to identify patterns with this display; more than 80% of participants preferred the vibration instead of electrical stimulation. No significant differences were found between the 2 vibrotactile displays. Ng et al [17,18] introduced the concept of vibrotactile displays for patient monitoring and reported that vibrotactile displays were superior in terms of *comfort* to electro-tactile displays. All later studies involving tactile displays used vibration instead of electrical stimulation. However, it is important to note that, ultimately, novel devices should be tested by the intended end users (experienced anesthesiologists) rather than nonclinicians, as was the case with these studies.

**Figure 8:**
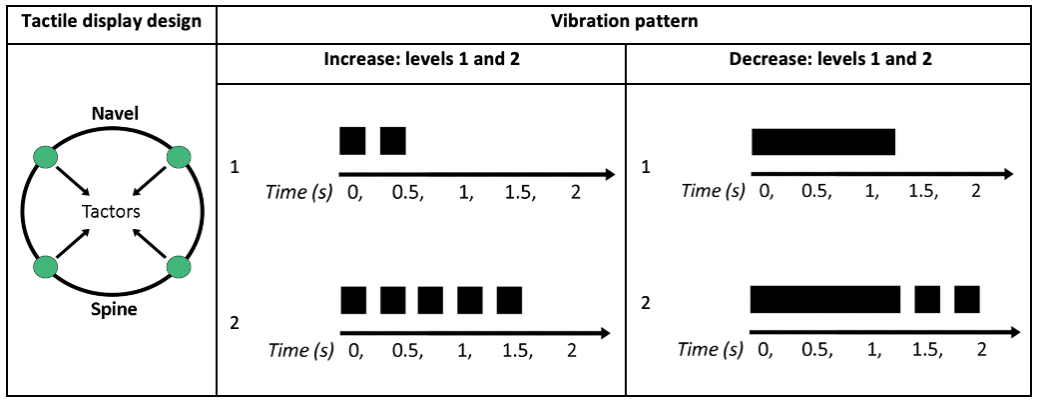
Tactile display worn around the waist. Each tactor represented a variable with 4 possible vibration patterns (permission to use the image obtained through RightsLink).

The display by Ng et al [19] worked in a similar manner to the previously discussed devices, but it was designed to be worn around the waist by anesthesiologists during an anesthesia procedure. It could monitor up to 4 variables simultaneously (Figure 8), with each tactor capable of generating 4 different vibration patterns. Therefore, a total of 16 different vibration patterns could be decoded by the clinician with this display. A total of 15 participants (certified specialist anesthesiologists and anesthesia residents) were asked to wear the tactile belt prototype and identify the IS being conveyed. The authors found that the IS identification was approximately 97% in low workload conditions and 93% in high workload conditions. The percentage of failed IS *detection* was 2% in low workload conditions and 17% in high workload conditions. Participants were reported to be satisfied with the user interface, but some participants expressed a preference for reducing the amount of information displayed. Although the study by Ng et al [19] demonstrated that potential end users could decode the information conveyed by the waist-worn tactile display, it is not possible to determine if these results indicate an improvement in patient monitoring, as this novel display was not tested against a PM.

**Figure 9:**
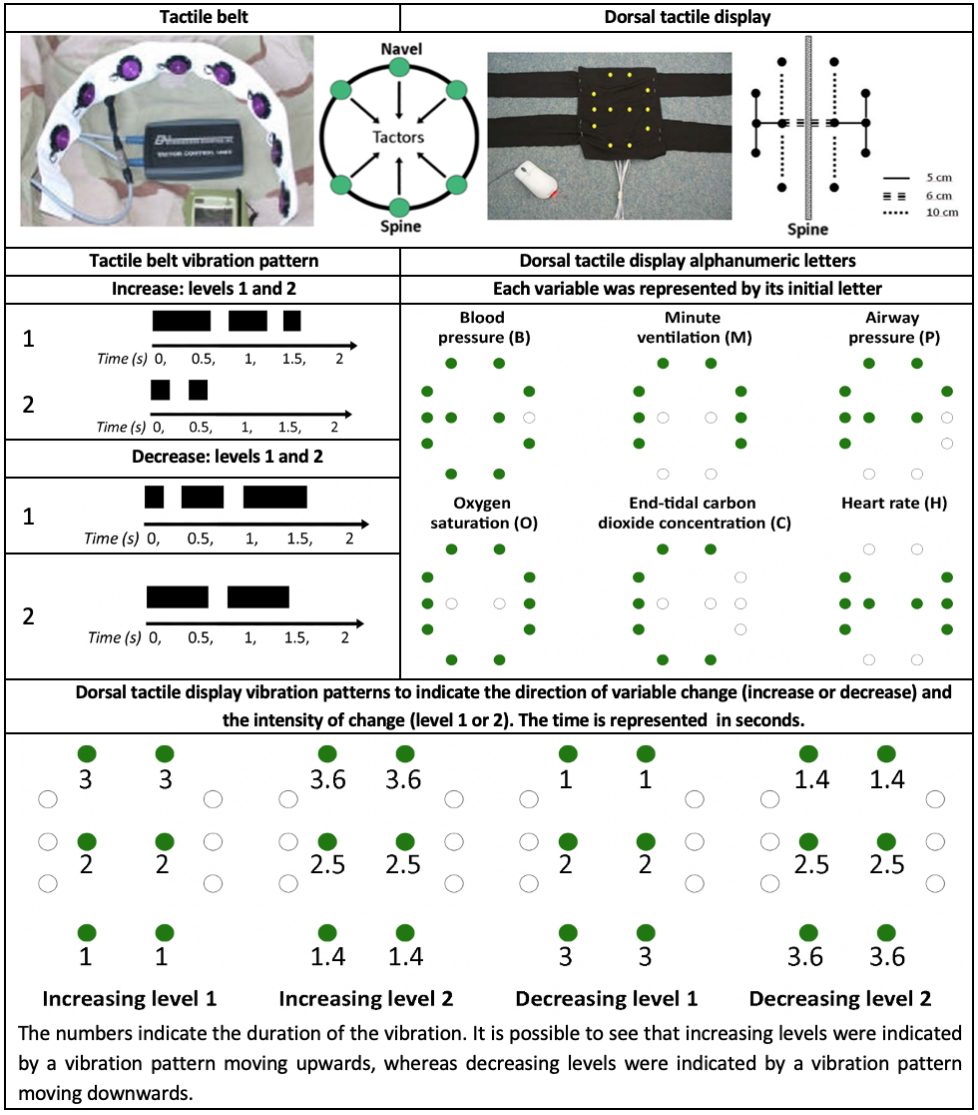
Tactile displays by Barralon et al [21]. Tactile belt worn around waist and dorsal tactile display positioned along the back. The tactile belt was designed to monitor 6 variables, each represented by a tactor with 4 possible vibration patterns. For the dorsal tactile display, each variable was represented by the tactors forming its initial letter. For each letter, the sequential locations were activated for 300 milliseconds, followed by a 700-milliseconds pause and a sequence of vibrations to indicate the level and direction of change (permission to use the image obtained through RightsLink).

The tactile device presented in Figure 8 was tested again in 2012 by Dosani et al [20]. This time, the tactile display was used to monitor pediatric patients undergoing general anesthesia. A total of 17 anesthesiologists (with a minimum of 3 years of experience with patient care) were asked to wear the tactile belt during anesthesia procedures. Once the patient’s physiological state was considered stable by the anesthesiologist, he or she turned on the tactile display, which then started receiving real-time vital sign data wirelessly from the PM. Every time that the belt vibrated, the anesthesiologist echoed their understanding of the tactile message into a computer. The device was evaluated in terms of *IS detection, IS identification,* and user *satisfaction.* In total, 530 alerts were delivered during the study, with 81.0% of them being decoded by the anesthesiologists (*IS detection*), and participants accurately identified 89.5% of the alerts (*IS identification*). In the study by Ng et al [19], as there was no control group in this study, it was not possible to determine if improved patient monitoring occurred. However, by testing this novel display with the desired end users during real patient monitoring, the authors acquired valuable usability information. Most participants indicated that they were comfortable wearing the tactile belt, whereas 6 participants reported that they would not be able to wear the tactile belt for a full workday. Clinicians reported that the mental process of decoding of messages became easier, with less mental effort, the longer the device was used, highlighting the importance of extended exposure to devices before testing. Barralon et al [21] compared 2 tactile display prototypes: a tactile belt to be used around the waist and a dorsal tactile display with an array of tactors located along the spine (Figure 9). The tactile belt and dorsal tactile display could monitor 6 physiological variables. Each tactile represented a specific variable with 4 possible alerts to represent the direction of change of the variable (increasing or decreasing), and the magnitude of change in the variable was categorized as level 1 or level 2. This resulted in 24 different alerts (6×2×2) that could be conveyed using the devices. Using 28 participants with no medical background, it was found that dorsal tactile display was easier to learn than tactile belt. It took longer to display the message with dorsal tactile display alerts (mean of 4.3 seconds) than with tactile belt alerts (mean 1.3 seconds). Participants using the tactile belt had a shorter *response time* than those using the dorsal tactile display. When measured from the end of the IS, however, the *response time* was shorter when participants used dorsal tactile display than that when they used tactile belt. This reflects the impact of the IS duration on *response time*. However, no statistically significant difference was found regarding *IS identification* of both devices. As these novel displays were not compared against a PM with clinicians, further studies to assess the usability of tactile belt and dorsal tactile display in clinical settings with the intended users would be desirable.

**Figure 10:**
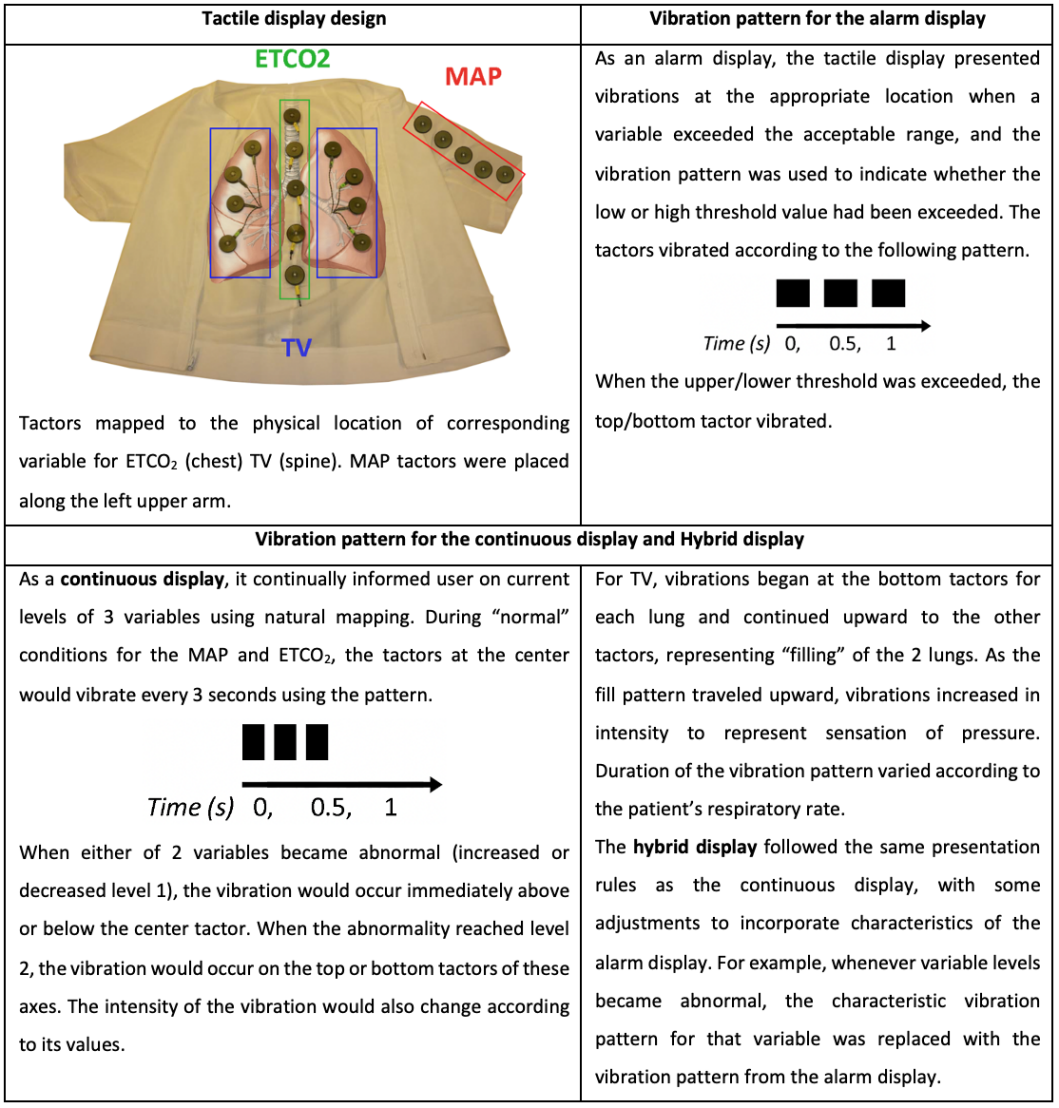
Tactile display by Ferris and Sarter [22]. The vest could be configured in 3 different modes: alarm, continuous, and hybrid display (image created based on the concepts presented in the paper and Ferris’ PhD dissertation).

Ferris and Sarter [22] developed a tactile display to monitor 3 variables. As shown in Figure 10, the apparatus had 3 different display modes: *alarm display*, *continuous display,* and *hybrid display*. The alarm display worked in a similar manner to the tactile displays previously discussed. The continuous and hybrid displays were 2 new concepts for tactile displays, which had not been tested before. The differences between these 3 display modes are detailed in Figure 10 (image created based on the concepts presented in the paper and in Ferris’ PhD dissertation [23]).

**Figure 11:**
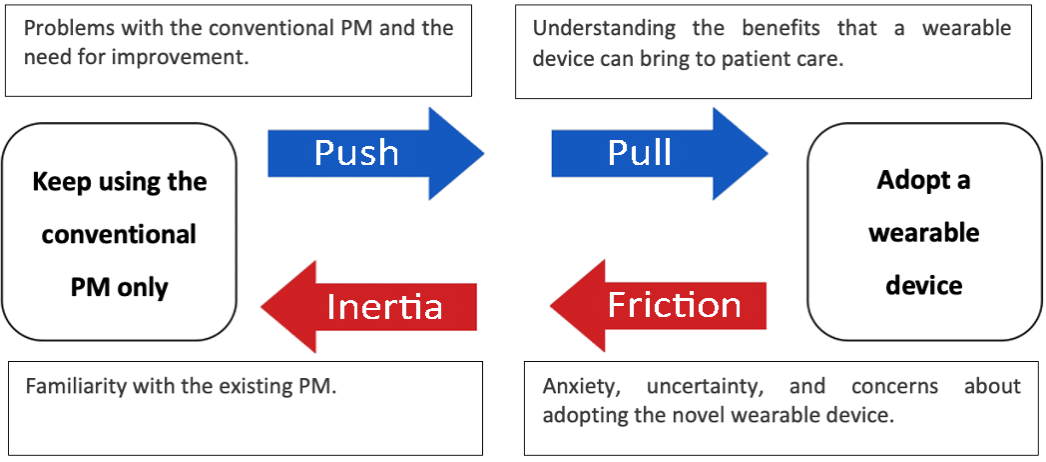
Balance of forces acting on the decision making of the clinicians when deciding if they should augment the patient monitor (PM) with a wearable display for critical care or continue only using the PM. Diagram adapted from the concept presented in “The Science of How Customers Buy Anything,” by Maurya [24].

In this study, 16 anesthesiologists were asked to (1) complete each scenario (containing at least 50 tasks each) as quickly as possible and (2) maintain the monitored variables within acceptable levels. The authors found that the *event detection time*, *event correction time*, and *multitasking performance* were statistically significantly improved when using the tactile displays compared with the PM. For instance, the mean event detection time was 56.4 seconds with the PM, 28.1 seconds with the alarm display, 26.8 seconds with the continuous display*,* and only 14 seconds with the hybrid display. No statistically significant differences were found for task completion time between displays. Despite the hybrid display’s better performance, participants felt that the alarm display and the PM display supported multitasking performance better. The authors suggest that this may be because of the display’s novelty and that participants would be inclined to choose interfaces they were familiar with over new ones. In addition, the participants considered the continuous and hybrid display uncomfortable, which can invariably generate concerns. These factors are all part of the balance of forces acting on the clinicians when deciding if they should augment the PM with a wearable display for critical care monitoring or continue using a PM only. This concept is presented in a diagram (Figure 11) adapted from “The Science of How Customers Buy Anything” by Maurya [24].

This feedback reinforces the importance of incorporating more extended familiarization with the wearable display before testing (especially when the wearable display has a large number of new concepts to be learned) and making the wearable as comfortable as possible.

**Figure 12:**
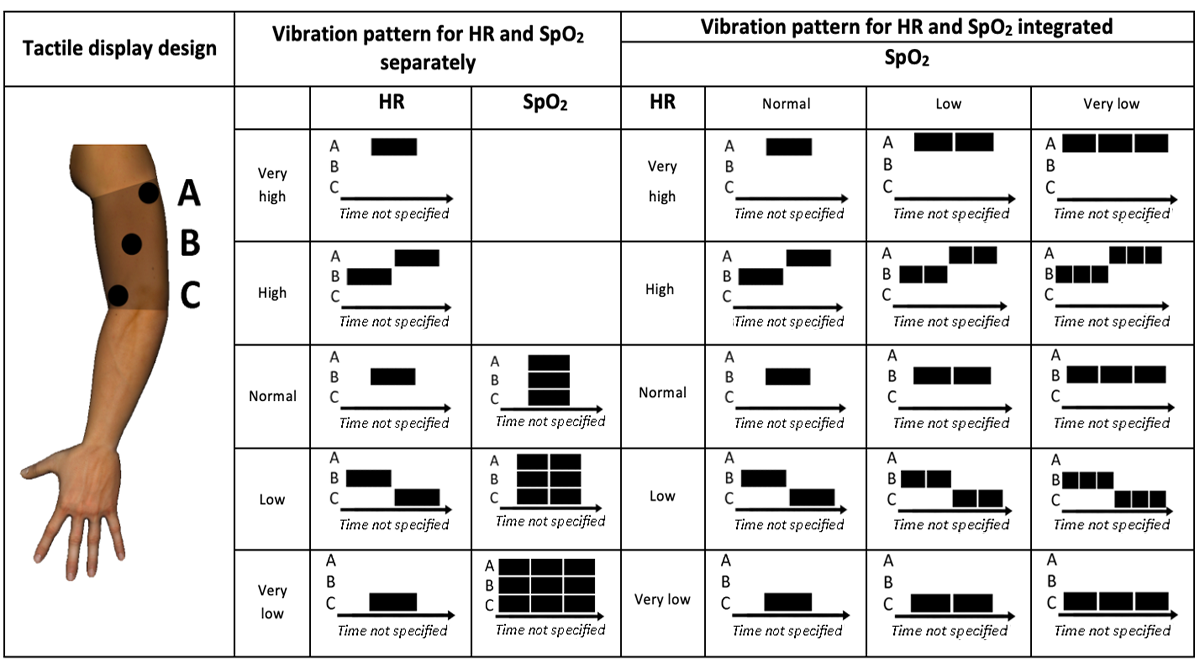
An elasticized tactile display sleeve on the upper arm with 3 tactors (A, B, and C) monitoring heart rate (HR) and saturation of peripheral oxygen (SpO_2_). This display could be used in 2 display modes: separate and integrated. In the separated display, the HR signal came first, followed by the SpO2 alert. In the integrated display, both alerts were displayed with a single alert (a model of the concept presented in the paper). H: heart rate; H: high; L: low; N: normal; VH: very high; VL: very low.

McLanders et al [25] investigated the use of tactile displays to continuously convey information from a pulse oximeter. In the study by McLanders et al [25], HR was continuously displayed as *very high*, *high*, *normal*, *low*, or *very low*, and the SpO_2_ was displayed as *normal*, *low*, or *very low*. As in the study by Ferris and Sarter [22], this reflected an attempt to communicate absolute values for the variables instead of communicating alarms only. As hospitals in the United Kingdom and Australia have adopted a *bare below the elbows* infection control policy since 2011, the authors determined that it was inappropriate to wear the tactile on the forearm and placed it on the upper arm instead. As shown in Figure 12, the tactile display could be used in 2 modes: *separated* and *integrated*. In the *separated* display, the HR alert was displayed first, followed by the SpO_2_ alert. In the *integrated* display, both variables were displayed using a single alert.

In a between-subjects study, 30 participants with no medical background were asked to test the prototype and to identify 5 ranges of HR and 3 levels of SpO_2_ in random sequences generated by a computer. Results showed no significant differences regarding *alert identification,* with participants recognizing over 90% of the changes in HR and SpO_2_ in both modes. There was a significant effect of display mode on the response time*,* with participants responding faster in the integrated mode. Regarding comfort, participants were moderately positive, with a mean score of 6.8 out of 9 on the comfort scale. The authors suggest that the use of wireless tactors may have contributed to the comfort of the devices, as they require less adhesive tape to secure the tactile display in place.

Cobus and Heuten [26] developed and tested a tactile display with the ICU nurse as the intended user. Unlike previous studies, the prototype used by Cobus and Heuten [26] was designed as an alarm system to inform the nurse of a possible risk to the patient, irrespective of which vital sign triggered the alarm, and was intended to reduce auditory alarm fatigue for nurses and patients by displaying the alarms silently. For this reason, only 3 vibration patterns were required to indicate 3 levels of urgency (eg, low, medium, and high). Similar to the study by McLanders et al [25], the display was placed in the upper arm for hygienic and safety reasons.

**Figure 13:**
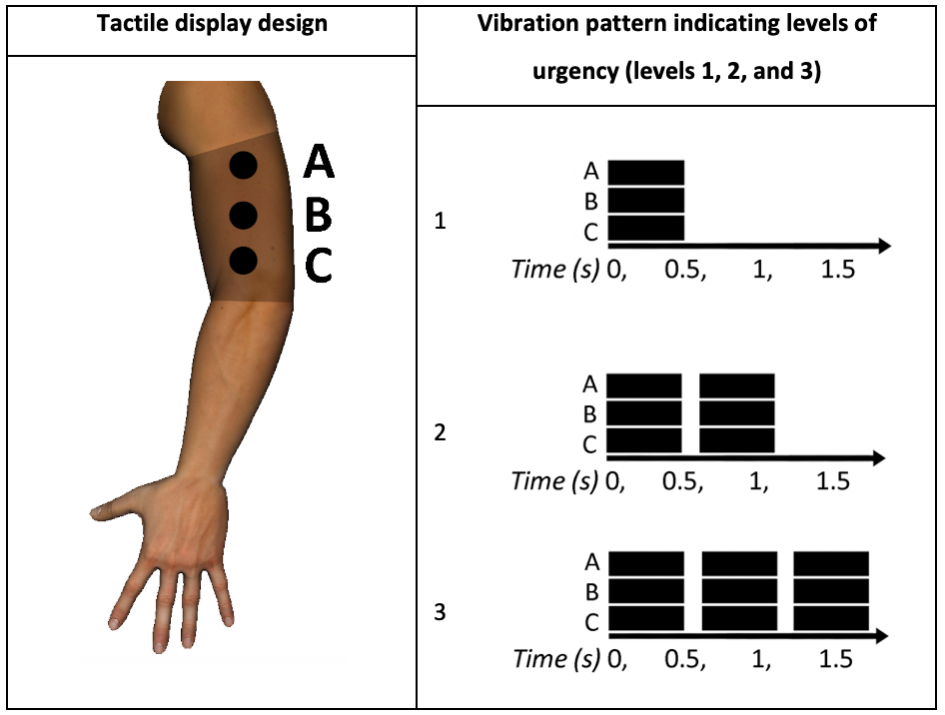
An elasticized sleeve on the upper arm holding 3 tactors (A, B, and C). Three vibration patterns indicated 3 levels of urgency, with the pattern repeating itself after a 800 milliseconds pause (a model of the concept presented in the paper by Cobus and Heuten [26]).

The prototype was tested initially by 12 participants with no medical background and then by 12 nurses to determine which alerts were better in terms of usability and comfort. The alert set shown in Figure 13 was chosen as most appropriate because of better IS identification. Although the chosen pattern was chosen as being most appropriate, it is worth noting that it may not be ideal for other tactile displays depending on the number of variables monitored, the tactile display position, and the context of use. Participants were also asked to complete a system usability scale (SUS) questionnaire to evaluate usability and a comfort rating scale (CRS) to evaluate the comfort of the prototype. The mean SUS was 95 (out of 100, which indicates very good usability), and a positive result for the CRS was also found. However, some participants reported that the device imposed arm movement limitations, revealing the importance of requiring the completion of physical tasks when testing these types of devices.

Burdick et al [27] investigated the effect of a multisensory alarm system that combined an auditory display with a tactile display. The multisensory display was compared with a unisensory display (auditory display only) regarding *alert identification* (identification of the variable, point of change, and direction of change). Interestingly, the auditory display used musical instruments to represent the variables: HR (drums), BP (piano), and blood oxygenation (guitar). Each variable had 3 levels of decrease, a normal level, and 3 levels of increase. The different levels were represented by changes in the timbre of the respective instrument. In the multisensory display, the different levels were also represented by a tactile display, where the auditory information was translated into vibration with equal rhythm and amplitude. Testing with nonmedical participants revealed that participants were better able to identify alerts when using the multisensory display. The authors commented that multisensory display might relieve auditory alarm fatigue in critical care.

**Figure 14:**
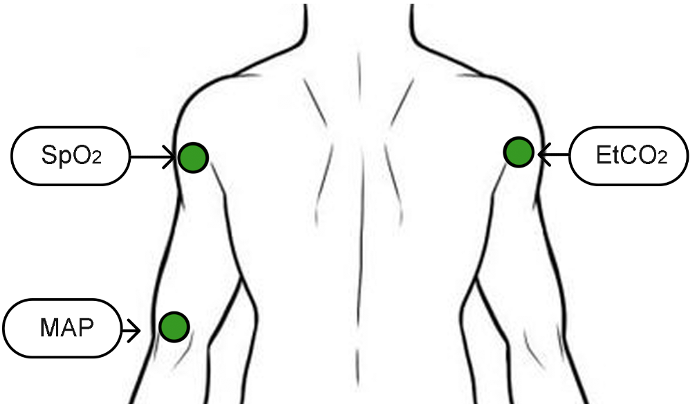
Tactile display, 3 types of alerts used for each variable: increasing, decreasing, and normalizing using 8 consecutive vibrations (500 milliseconds in duration). If the variable value was increasing, the intensity of the vibration increased during the 8 consecutive vibrations, and if the variable value was decreasing, the intensity of the vibration decreased. When the variable value was normalizing, the intensity was kept constant (a model of the concept presented in the paper by Gomes et al [14]). EtCO_2_: end-tidal carbon dioxide partial pressure; MAP: mean arterial blood pressure; SpO_2_: oxygen saturation.

The tactile display studies discussed varied significantly in design (eg, variables monitored, location of the display, and vibration pattern). This reveals a lack of consensus on the best tactile display design for critical care medicine. Gomes et al [14] aimed to address this literature gap by conducting 2 experiments. In the first one, the authors evaluated the usability of the 3 main parameters of tactile displays: intensity of vibration, vibration pattern, and position of tactors. In total, 22 health care professionals were asked to test a tactile display, similar to the one described in Figure 13, and answer a set of usability questions about the alerts presented. On the basis of the results of the first experiment, Gomes et al [14] then designed the tactile display presented in Figure 14. Like Ferris and Sarter [22], Gomes et al [14] understood that the use of mapping can be an effective way to improve the device’s usability. However, instead of mapping the location of the tactors to the physical body location of the corresponding variable, the tactors were mapped to the display locations in a PM. For instance, SpO_2_ and mean arterial BP values were displayed on the left side of the PM used by the participants, with SpO_2_ located above mean arterial BP, whereas end-tidal carbon dioxide partial pressure (EtCO_2_) was shown on the right side.

A total of 19 participants (9 attendings, 7 residents, and 3 certified registered nurse anesthetists) tested the developed tactile display and identified the presented cues with a response accuracy of ≥90%.

A summary of the results of the studies involving tactile displays is presented in Appendix 2 [14,17-22,25-35]. It should be noted that it is sometimes difficult to compare the same metrics across different studies, as study design differences can make comparison meaningless. Most tactile displays reviewed were prototype devices developed to determine the feasibility of using the tactile sense to convey the patient’s physiological state. For this reason, most authors focused on the subject’s capability to detect, identify, and respond to an IS produced by the tactile display. Therefore, the performance metrics most evaluated in the studies involving tactile displays were *IS detection; IS identification; response time;* and some usability metrics such as *comfort*, *satisfaction*, and general *usability*. These metrics were chosen as they were used by most studies reviewed. For the purpose of uniformity, the values of usability metrics that were evaluated using scales (eg, SUS, Likert-type scales) were converted to a scale of 1 to 7, with 1 being very negative and 7 being very positive (eg, a 3 in a 1-5 scale became a 4 in this 1-7 scale).

### Head-Mounted Displays

**Figure 15:**
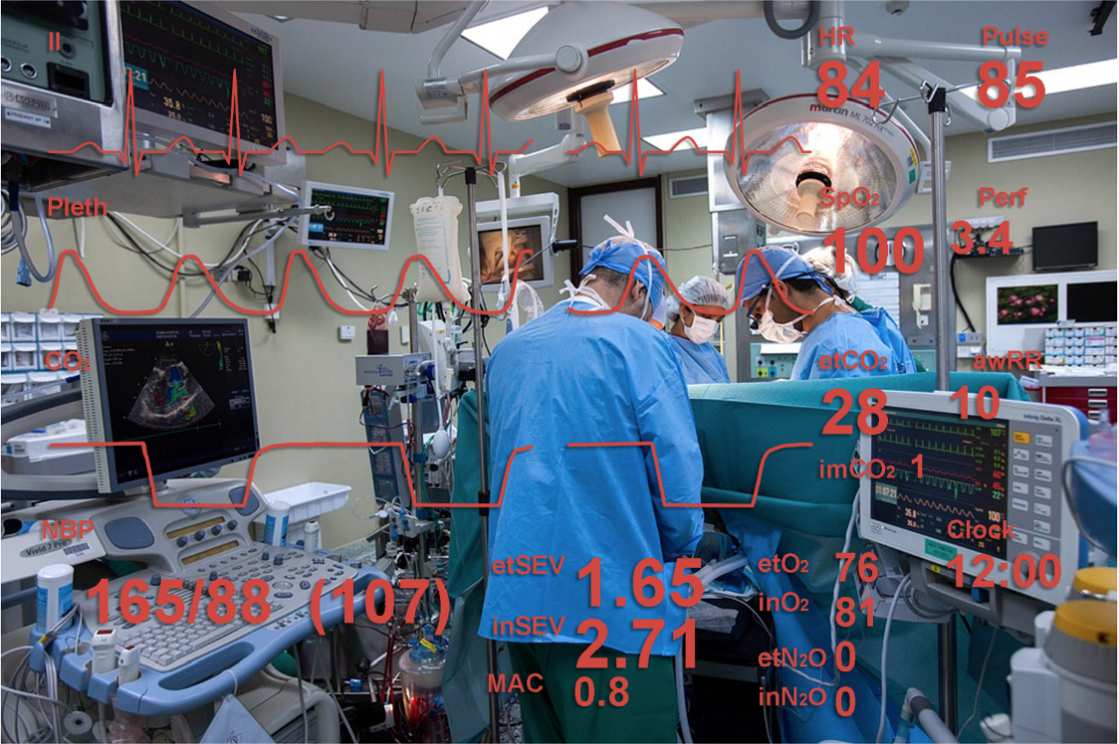
A visual representation of the view of an anesthesiologist wearing the head-mounted displays presented in Liu et al [29]. awRR: airway respiratory rate; CO_2_: carbon dioxide; EtCO_2_: end-tidal carbon dioxide partial pressure; etN_2_O: end-tidal nitrous oxide concentration; etSEV: end-tidal sevoflurane concentration; HR: heart rate; imCO_2_: inspired minimum CO_2_; inN_2_O: inspired nitrous oxide concentration; inSEV: inspired sevoflurane concentration; MAC: minimum alveolar concentration; NBP: noninvasive blood pressure; SpO_2_: oxygen saturation.

Sanderson et al [28] evaluated the advantages and disadvantages of HMD for anesthesiologists compared with traditional auditory displays. They asked 16 participants (7 consultants and 9 residents) to supervise the activities of a resident (an actor) during anesthesia under 4 display conditions: *visual* (PM plus variable-tone pulse oximetry [control condition]), *HMD* (*visual* plus *HMD*)*, audio* (*visual* plus respiratory sonification and BP audio IS) and *both* (*HMD* plus *audio* conditions). The HMD presented the vital signs in a manner similar to that shown in Figure 15, but without the traces. Significantly more events were detected with *audio* and *both* conditions compared with the *visual* condition only. However, no statistically significant differences were found when comparing HMD and visual conditions. No differences were found regarding the *event detection time* for all displays. When asked about their preferences, most participants (83%) liked the easy availability of information on the HMD, but 56% disliked comfort aspects such as weight and size and referred to experiencing headaches.

Liu et al [29] investigated if HMD during anesthesia procedures would worsen *inattentional blindne*ss, for example, the HMD may put the anesthesiologist in a state of immersion resulting in him or her missing salient, unexpected events that they would otherwise not miss. This issue has been reported in other domains such as aviation [36]. In the study by Liu et al [29], the variables were displayed in the same format as in the PM, with the waveforms presented on the left and digital numeric values on the right. However, all the variables were displayed in red instead of a color-coded format frequently used in PMs (Figure 15). Two experiments were conducted with an HMD connected to a PM. In the first experiment, 12 anesthesiologists were asked to perform surgical simulation scenarios in 3 different contexts: focal depth of the HMD near, focal depth of the HMD far, and no HMD. It was found that *event detection* and *event detection time* were not significantly affected by the use of HMD (near or far focus), suggesting that *inattentional blindne*ss may not be a major cause of concern. Importantly, it was found that participants spent more time looking toward the patient rather than the monitor when using the HMD (near or far focus). In general, participants found the non-HMD the easiest and preferred condition. Participants liked that the HMD gave them the capability to monitor the patient’s vital signs, irrespective of the direction of their gaze or their location in the operating room. Nonetheless, they disliked the weight or size of the HMD and associated computer equipment and the difficulty of focusing on the HMD, which caused eye fatigue. Participants also preferred the near-focus setting when using the HMD.

In the second experiment conducted by Liu et al [29], the goal was to examine whether or not HMDs would be useful if anesthesiologists were operationally and physically constrained (PM behind them, forcing participants to rotate their trunks to observe PM). Under these circumstances, participants using the HMD significantly improved *event detection time* in 2 of the 3 scenarios (light anesthesia and hypovolemia). However, in the excess sedation scenario, *event detection time* was significantly lower. Once again, participants spent more time looking at the patient rather than at the monitor when using the HMD during this experiment. Participants rated the scenarios in which they used the HMD, as being less busy, easier for monitoring patients, and faster for detecting vital sign changes than those scenarios with the PM only. Once again, participants liked not having to turn around to look at the PM but felt somewhat uncomfortable using the HMD because of the weight and size of the device and its associated equipment. The investigation conducted by Liu et al [29] revealed that, by reducing the number of required neck rotations by the anesthesiologist, HMDs had ergonomic benefits. In addition, by keeping the patient in his or her visual field for longer, the anesthesiologist is potentially less likely to miss a critical clinical event (eg, increase in skin pallor). Therefore, HMDs could not just increase comfort but also improve patient safety. In a 2010 paper, Liu et al [30] investigated if using HMD during an anesthesia procedure would result in 6 anesthesiologists spending more time looking at the patient and less time looking at the monitor when delivering anesthesia to 6 real patients, alternating between the experimental condition (PM plus HMD) and control condition (PM plus HMD equipment without the monocle that displayed the vital signs). In the experimental condition, participants spent less time looking toward the workstation and more time looking toward the patient and the surgical field. Regarding comfort and satisfaction, although participants did not have significant positive or negative views about the HMD, they raised the same issues regarding the weight and bulk of the HMD, as in the study by Liu et al [29].

**Figure 16:**
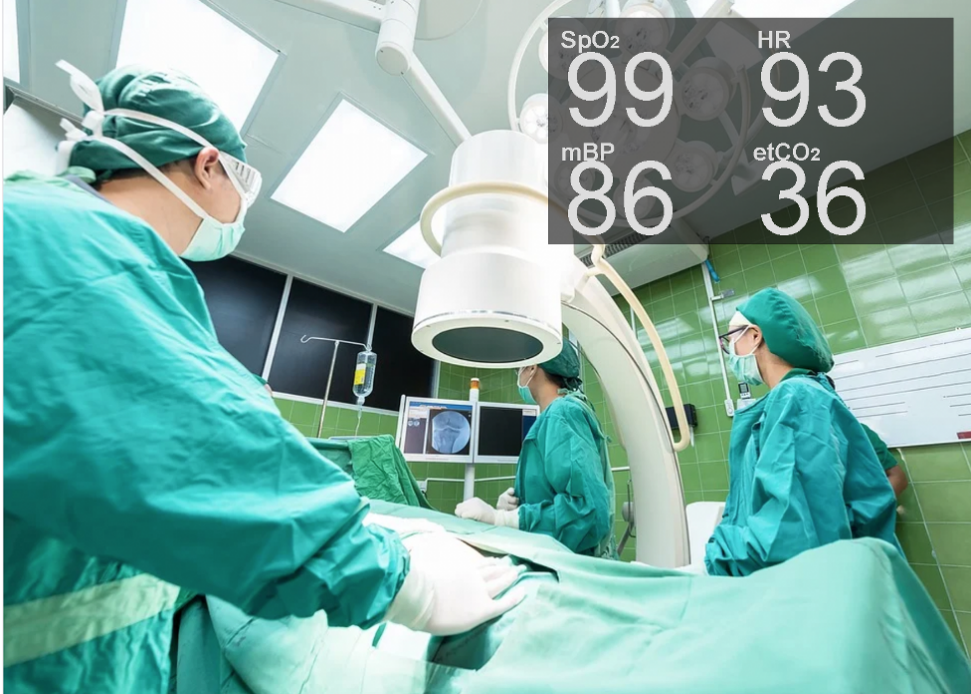
A mock-up of the views experienced by anesthetists in the study by Drake-Brockman et al [37] (a model of the concept presented in the paper). EtCO2: end-tidal carbon dioxide partial pressure; HR: heart rate; mBP: mean blood pressure; SpO2: oxygen saturation.

Three researchers evaluated the usability of Google Glass for patient monitoring. Drake-Brockman et al [37] evaluated the acceptance of Google Glass by 40 anesthesiologists in a pediatric anesthesia context. As shown in Figure 16, the interface design was composed only of the digital values for 4 variables: SpO_2_, HR, BP, and ETCO_2_.

An important finding was that the HMD comfort issues identified by Liu et al [29] were rectified with Google Glass. Participants reported that the device was comfortable to wear (90%), easy to read (86%), and not distracting (82.5%). Moreover, 76% of participants reported that they would use it again, and 58% indicated that they would recommend the device to a colleague. Anesthetists with less experience (generally younger) were less averse to wearing the device in front of patients (78%) than more experienced ones (43%).

**Figure 17:**
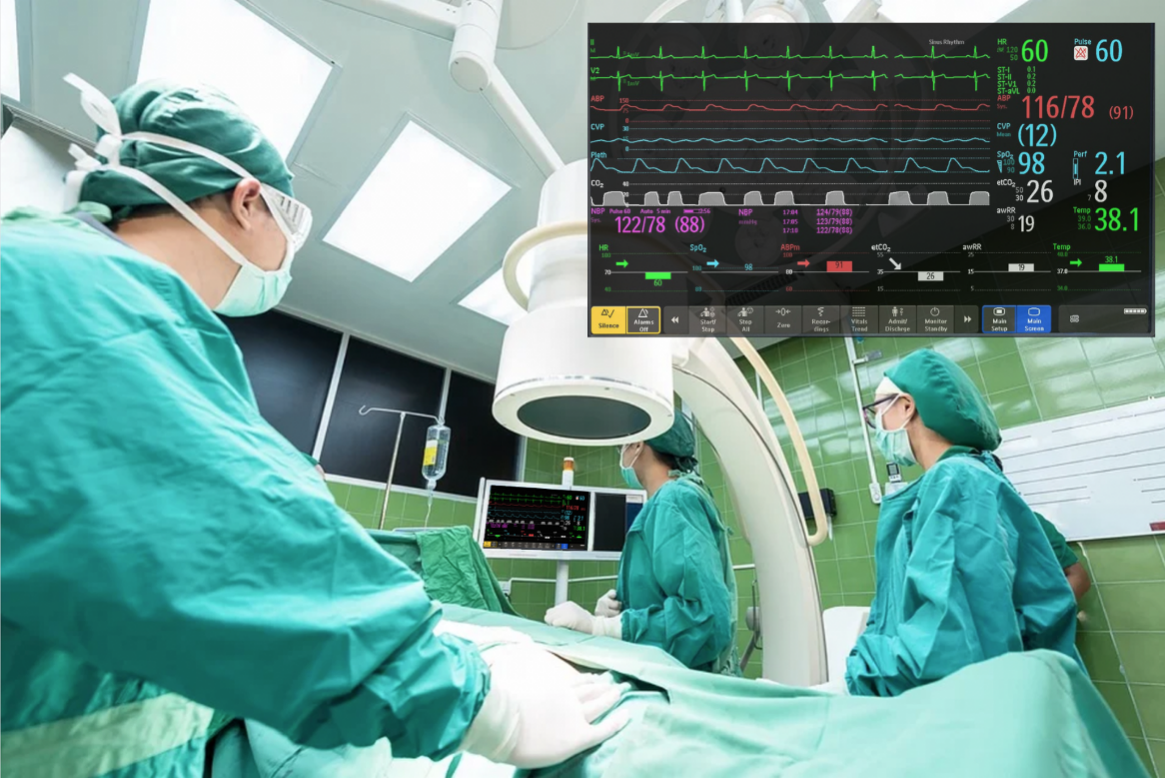
Representation of participant’s view when wearing the Google glasses (a model of the concept presented in the paper by Liebert et al [31]).

Liebert et al [31] also used Google Glass to display patient vital signs during a medical procedure. In the display used in the study by Liebert et al [31], the entire PM screen was visible in the top-right corner of the glasses (Figure 17) instead of only a subsection, as in the study by Brockman et al [37]. In total, 14 surgical residents participated in 2 simulated scenarios: a thoracostomy tube placement and a bronchoscopy, interacting with a high-fidelity mannequin (Laerdal SimMan 3G). Participants in the experimental group (1) recognized the event (hypotension) faster, (2) made significantly fewer glances toward the PM, and (3) spent significantly less time looking at the PM. Similar results were found in the bronchoscopy scenario.

Most participants agreed that the device was easy to use (93%), improved their *situation awareness* (SA; 64%), helped to monitor vital signs (86%), and had the potential to improve patient care (85%). In addition, 86% of participants would consider using Google Glass in their future clinical practice.

**Figure 18:**
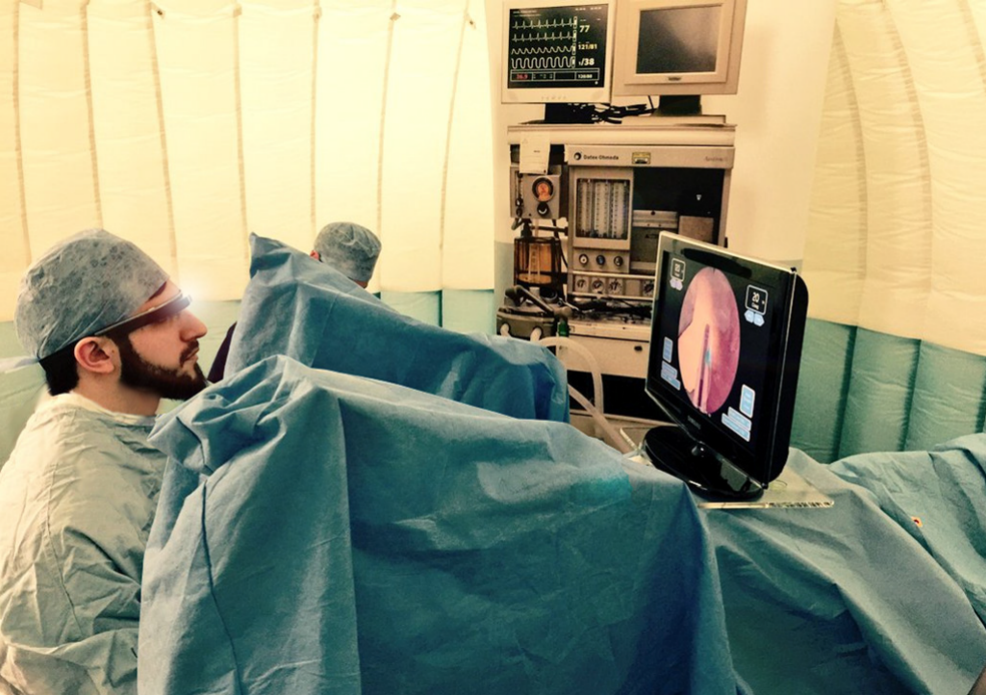
An urologist wearing Google Glass during a GreenLight prostatectomy. The patient monitor is visible in the top of the figure. During prostatectomy surgery, monitoring of patient’s vital signs is primarily the responsibility of the anesthesiologist; however, Iqbal et al [32] argued that Google Glass enabled the urologist to focus on the surgical site without having to discuss vital signs with the anesthesiologist (permission to use the image obtained through RightsLink).

Iqbal et al [32] evaluated the acceptance and performance of Google Glass with urologists. The interface designed for the experiment and the variables presented in the display were not provided. They asked 37 subjects (24 medical students, 8 urology surgical trainees, and 5 consultant urologists) to perform a simulated surgery (laser prostatectomy), initially using only the PM and then using the PM in conjunction with Google Glass. *Response time* to the vital sign changes was significantly shorter when using the Google Glass (mean of 35.5 seconds) compared with PM only (51.5 seconds). There may have been an order effect, as all participants performed the control simulation first, followed by the experimental simulation using the same scenario. Most participants reported that Google Glass increased their awareness of vital signs and that they would use the device during surgical procedures. Participants who already wore prescription glasses and were left-handed reported discomfort wearing the device, as it needs to be placed on top of the user’s glasses and only displays data to the right eye. The authors identified battery life and comfort issues for prescription glass users as potential barriers to its adoption into clinical practice. Figure 18 shows one of the study participants wearing Google Glass during a “GreenLight” simulated prostatectomy.

**Figure 19:**
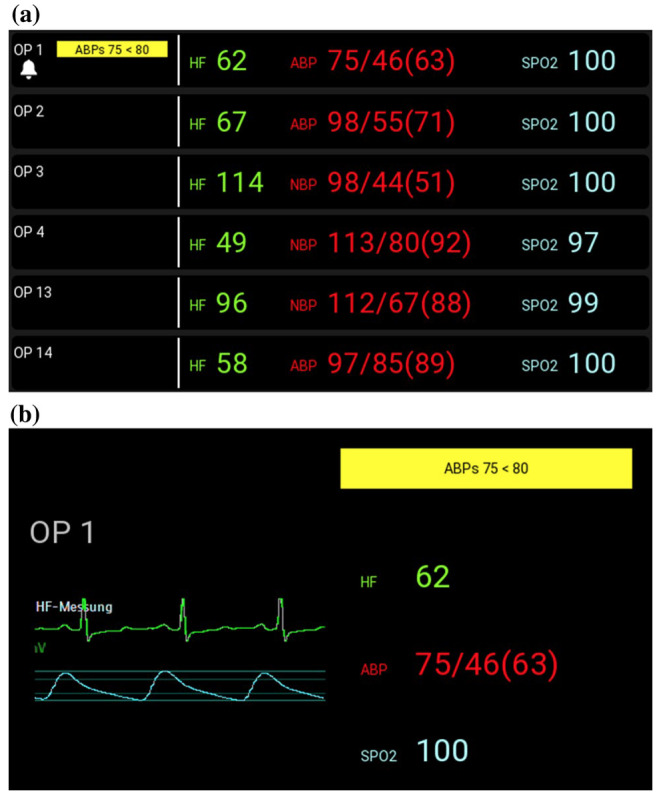
Schlosser et al's [33] display, as presented in the head-mounted displays (HMDs) prototype. (A) alarms are displayed on the left side of the screen, and the digital values for heart rate, blood pressure, and saturation of peripheral oxygen are displayed on the right side. (B) A second screen of Schlosser’s display was designed to present more details (such as a snapshot of the electrocardiogram curve) for one specific patient. In addition to the visual alarms, auditory alarms were displayed on the HMDs via bone conduction. To interact with the device, a button on the HMDs had to be pressed to cycle through the patients. (Permission to use the image obtained through RightsLink.) ABP: arterial blood pressure; HF: heart rate; NBP: noninvasive blood pressure; OP: operating room; SpO_2_: oxygen saturation.

Schlosser et al [33] proposed the use of HMDs by anesthesiologists for vital sign monitoring of multiple patients simultaneously in operating rooms. Schlosser et al [33] used the Vuzix M300 (Vuzix Corporation) glasses and developed the user interface through a user-centered design process. The prototype (Figure 19) was connected to the PM network and could display a subset of the PM vital sign data for up to 6 patients and reproduce the alarm sounds for the different patients. A total of 8 anesthesiologists were asked to monitor 6 patients simultaneously for 3 hours while wearing the HMD and for 3 hours without the HMD. Schlosser et al [33] reported that the number of alarms detected by the anesthesiologists was significantly higher when using the HMD (66.7% vs 7.1%). This is a very significant result. With regard to the usability of the HMD, participants indicated satisfaction in terms of readability, interface structure, and navigation. However, they reported that the HMD interfered with the tie-on laces of the surgical mask. In addition, 4 of the 8 participants considered the HMD too heavy (55 g) and too big. Another important issue raised was that participants considered the HMD alarms distracting when they were performing activities that required focus.

**Figure 20:**
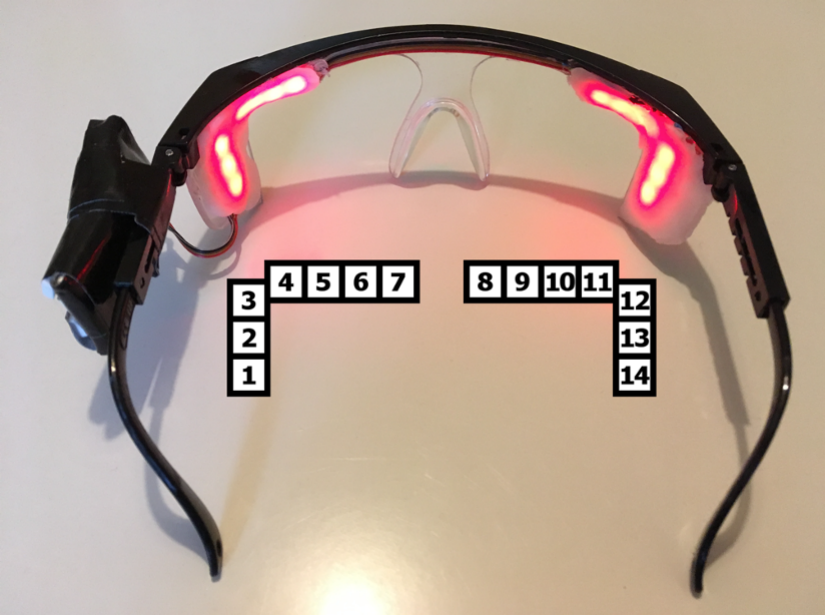
Cobus and Heuten’s [26] head-mounted displays displaying a high-priority alarm. All light-emitting diodes (LEDs) were activated simultaneously for the alarms. The peripheral light followed the alarm colors commonly used by patient monitors. Red was used for high-priority alarms, yellow for low-priority alarms, and blue for technical alarms (alarm indicating a technical problem, eg, sensor not connected).

**Figure 21:**
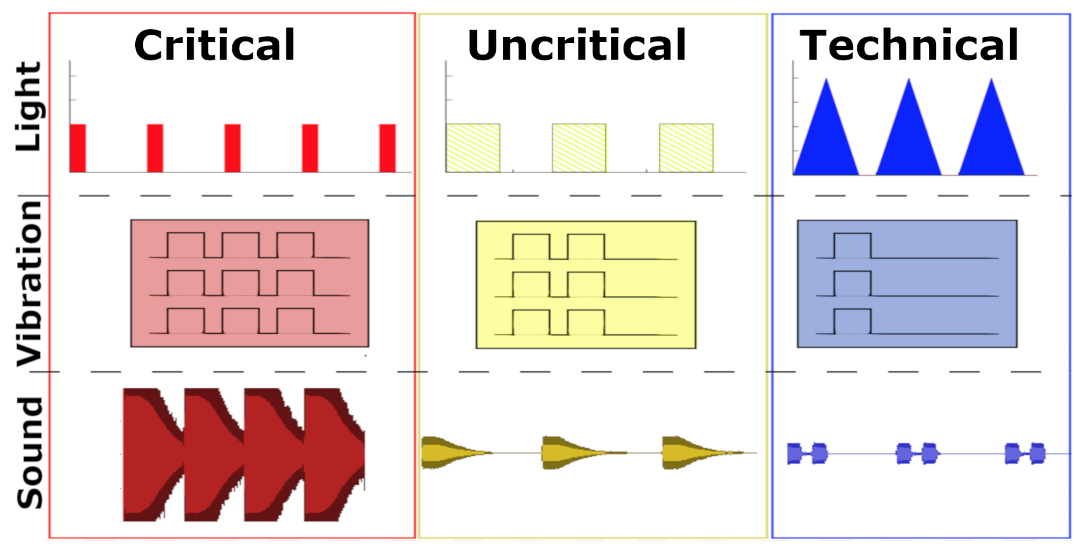
Lights, vibration, and sound patterns generated by the “peripheral light, tactile, and auditory” displays, respectively by Cobus and Heuten [26].

Cobus and Heuten [26], in addition to the upper arm tactile display presented in the previous section, designed an innovative way to silently alert ICU nurses of PM (silenced) alarms. The prototype wearable, presented in Figure 20, uses peripheral lights of 3 different colors to indicate a technical, low-priority, or high-priority alarm. Other wearable displays to present silenced PM alarms were also investigated: a wearable *audible* display that transmitted the PM alarms via bone conduction speakers using the same sounds used by the PMs and a *tactile* display that vibrated when an alarm occurred. Figure 21 depicts the light, vibration, and sound patterns generated by the different elements of the wearable*.*

The research team asked 12 ICU nurses to identify several alarms using the *peripheral light*, *audible*, and *tactile* displays individually versus the PM audible alarm. It was found that participants made significantly more errors with wearable audible alarms and PM audible alarms. However, participants reported that they were used to, when hearing the PM alarm sound, to look at the PM display to identify the alarm’s cause. This indicates that as the purpose of the wearable’s display is to augment the PM, it would have been desirable to have the PM as part of the test scenario. In terms of IS identification time, although participants were faster when using the peripheral lights display in comparison with all others, participants raised concerns regarding the brightness of the lights of the peripheral light display, indicating that it was exhausting for the eyes and prone to triggering headaches.

**Figure 22:**
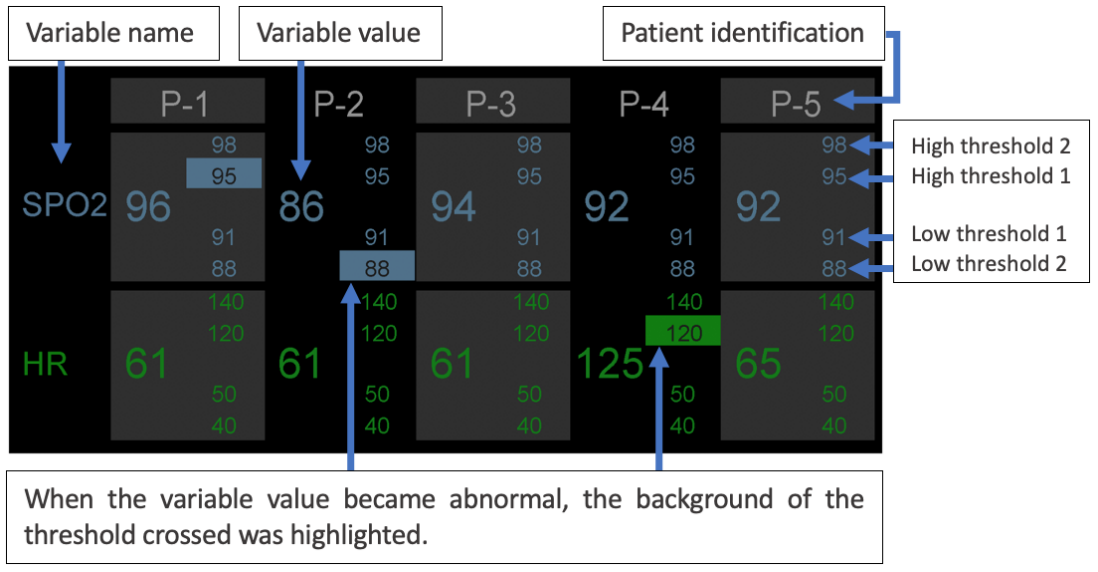
Information on the head-mounted displays by Klueber et al [34]. In this scenario, patients P-1, P-2, P-3, and P-4 have abnormal variables. Patient P-1 has exceeded the first high threshold for saturation of peripheral oxygen (SPO_2_; 95%), and patient P-2 dropped below the second low threshold for SPO2. Patient P-3 has exceeded the first high threshold for heart rate (a model of the concept presented in the paper). HR: heart rate; P: patient; SpO_2_: oxygen saturation.

Klueber et al [34] evaluated 2 displays designed for multiple patient monitoring: an HMD and an *auditory* display. The Vuzix M100 (Vuzix Corporation), which is an opaque monocular HMD that includes an earpiece for audio, was used for both displays. The design of the HMD interface can be seen in Figure 22. Using the Vuzix M100 earpiece, the auditory display presented time-compressed recordings of 500 milliseconds duration, verbalizing the variable name and variable level. For example, to convey that the values for SpO_2_ and HR were normal, the auditory display verbalized *sat normal pulse normal*. The pitch and tone of the verbal cues were different depending on the severity of the patient’s state. A total of 57 undergraduate students were randomly assigned to test 1 of the 3 groups: visual HMD, auditory HMD, or combined HMD. In terms of IS identification*,* participants using combined HMD or visual HMD alone performed significantly better than participants using auditory HMD. When asked to do a parallel activity (a precision computer task), which required constant visual attention, participants using the combined HMD performed better than participants using the visual HMD. Nonetheless, further studies involving clinicians are necessary to assess the *suitability* of these displays in critical care settings.

**Figure 23:**
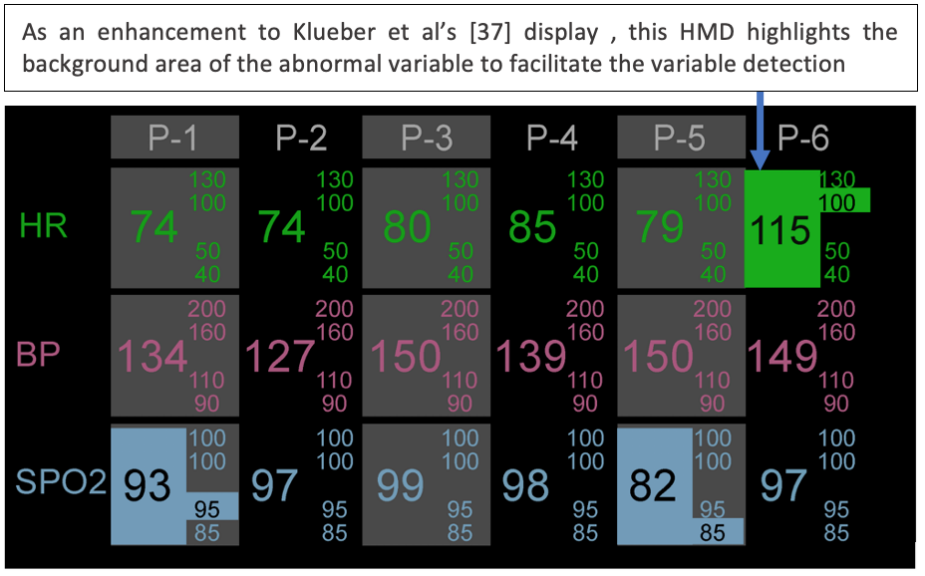
Continuous streams of patient data were presented on the head-mounted displays for up to 6 patients by Pascale et al [35]. The display was similar to the one from the study by Klueber et al [34], with the difference that this display also monitored blood pressure and abnormal values had their background highlighted. In this example, we can see that patients P-1, P-3, P-5, and P-6 have abnormal variables (a model of the concept presented in the paper). BP: blood pressure; P: patient; SpO_2_: oxygen saturation.

Pascale et al [35] also evaluated the use of HMD for continuous monitoring of multiple patients augmenting PM alarm sounds (Figure 23). In the first experiment with 76 undergraduate participants, it was verified that the PM alarms+HMD group responded to the alarms statistically significantly faster than participants in the PM alarm–only group. In the second experiment, the focus was to investigate if HMDs would improve SA. The authors developed an advanced auditory display (referred to as *notifications*) as a replacement for the PM alarms and tested it in conjunction with an improved version of the HMD. The notification display sounded in the earpiece of the HMD (Vuzix M100) when a variable value threshold was crossed, including when a variable value moved from abnormal to normal.

A sound was played for each patient, based on their status, in the same order as the visual display. Therefore, notifications consisted of 6 consecutive sounds. The notification could be one of three 500 milliseconds tones: (1) a low-pitched beep with no tremolo indicating normal, (2) a medium-pitched beep with slow tremolo indicating that the first threshold was crossed for at least one vital sign for that patient, and (3) a high-pitched beep with faster tremolo indicating that the second threshold was crossed for at least one vital sign. In total, 13 second- and third-year nursing students participated in the experiment and tested the 3 display modalities: (1) PM alarm, (2) PM alarm+visual HMD, and (3) PM alarm+visual and auditory HMD. It was verified that participants answered the SA questions significantly more accurately, obtained higher scores on the ongoing patient assessment, and reported lower workload when they used the display modalities (2) and (3) in comparison to modality (1). Additionally, when using display modality (3), participants answered the SA questions significantly more accurately than when using modality (2).

A summary of the results of HMD studies is presented in Multimedia Appendix 2. Most studies were performed with experienced clinicians as test subjects, which allowed researchers to test if these devices could improve clinicians’ detection of clinical events during simulations. For this reason, *event detection, event detection time*, and *response time* were the main performance metrics used in these studies. As each study used different test events during the experiments and had different study designs, it is difficult to compare results across studies. However, most studies included the PM (screen or auditory alarm system) as a control display, which provides us with an opportunity to evaluate how the HMDs compared with the PM under the same test conditions.

### Smartwatches

**Figure 24:**
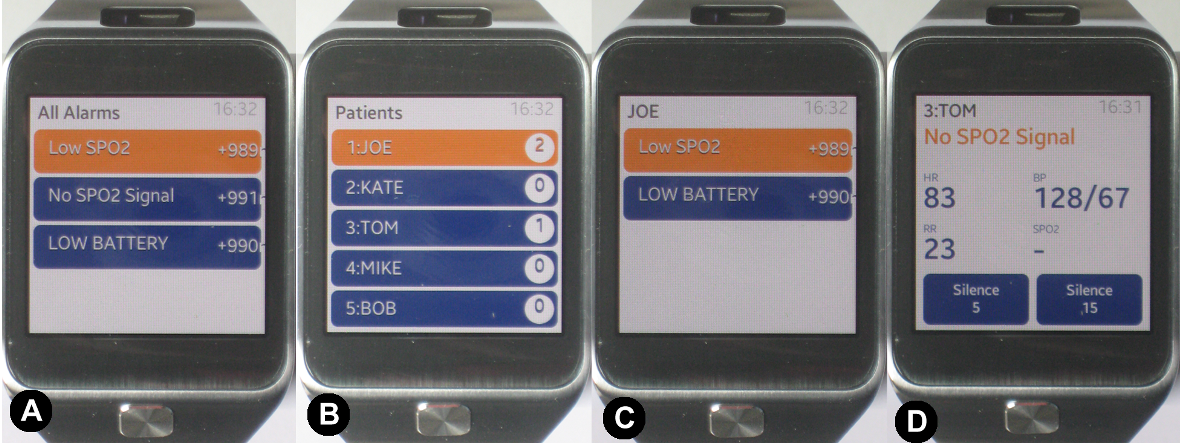
Alarm system app running in a smartwatch with 4 screens by McFarlan et al [38]: (A) list of all alarms related to any patient monitored by the nurse, (B) list of the 5 patients monitored by the nurse, (C) list of all alarms for a selected patient, and (D) patient view with the alarm message on the top and the values for the patient’s vital signs. The blue background indicates silenced alarms, and the orange background indicates alarms that are not silenced.

Another wearable that is starting to be explored for use in critical care patient monitoring is the smartwatch. McFarlan et al [38] tested the applicability of nurses using smartwatches when monitoring multiple patients simultaneously. A smartwatch app was developed to support ICU nurses to respond to alarms quickly. The smartwatch displayed alarms and patient vital signs and interacted with the actual PM, silencing it when an app button was pressed. The screens from the smartwatch app and explanation of the interface can be seen in Figure 24.

In total, 16 nurses undertook highly realistic multitasking within a simulated clinical unit using patient mannequins. The outcome measure used in this study was response time. The nurses received information and instructions about the patients and were asked to use their clinical judgment in deciding how and when to respond to alarms and call button events. Testing involved 20 simulated patients and 4 nurses; each nurse was assigned randomly to 5 patients. The experiment was divided into 2 parts (randomized across nurses): 90 minutes in the control conditions (using the PM only) and experimental conditions (with the smartwatch and PM).

It was observed that nurses responded to the alarms significantly faster with the PM+smartwatch display, with a median difference of −6.14 minutes (cumulative response time for all alarms in the experiment for each nurse) in the response time to important alarms or alerts. It was reported that the smartwatch display did not interfere with nurses’ workflow. The smartwatch display gave the nurses the possibility of silencing the alarm without being near the PM and was rated positively in terms of usability; all nurses said they would use the system in real conditions.

## Discussion

### Tactile Displays

#### Overview of the Studies

Tactile displays were one of the first wearable devices investigated as a means to augment PMs in critical care medicine. This review found that tactile displays can potentially diminish the noise generated by PM alarms and enable the clinician to be alerted when the patient’s vital signs cross alarm thresholds, without having to avert their gaze from the patient toward the PM.

#### Tactile Device Location and Number of Monitored Variables

Regarding the ideal location of a tactile device on the clinician’s body, different authors had different design approaches. For example, for a small number of monitored vital signs, the forearm and wrist were initially found to be suitable locations [18], with more recent studies proposing the upper arm as a better location for hygienic purposes [25,26]. In the case of a higher number of monitored vital signs, the waist was identified as a suitable location because of the greater number of tactors, which must be accommodated [19-21]. Only 2 studies have tried *mapping* as a strategy to provide clinical information in a more user-friendly manner, reflecting best practices in usability engineering [39]. Ferris and Sarter [22] mapped the tactors’ location to the physical location of the corresponding variable, and Gomes et al [14] mapped the location of the tactors according to the position of the respective variables on the PM display.

Subjects wearing tactile displays with a higher number of monitored variables (consequently, a higher number of different IS) are likely to achieve lower IS detection and identification compared with subjects wearing tactile displays to monitor fewer variables. *Response time* also seems to be profoundly affected by the number of variables monitored, with participants monitoring more than 3 variables taking generally longer to respond to the IS than participants monitoring a maximum of 2 variables. Therefore, using tactile displays to monitor a large number of variables might not be desirable.

#### Usability and Ergonomics Aspects

Regardless of the tactile device’s positioning on the clinician’s body or the number of monitored variables, *comfort* was a recurring theme, with several participants reporting discomfort or lack of mobility when wearing the displays [17,22,26]. It should be noted that the evaluated devices were prototypes fabricated in a research setting, and thus, the devices may not have been optimized from a design or fabrication perspective. A commercial product that incorporated these concepts would benefit from miniaturization using state-of-the-art manufacturing techniques and a full industrial design intervention and would thus be expected to overcome some of these usability issues. For instance, by using new technological components (wireless tactors), McLanders et al [25] reported fewer discomfort issues than previous studies. In conclusion, researchers must keep in mind that comfort has a significant impact on the perception of end users of a wearable device. The user may be reluctant to adopt a novel wearable technology that would enhance their performance if they do not feel comfortable wearing it.

#### Performance Metrics

As the purpose of these devices is to augment critical care patient monitoring by increasing a clinician’s ability to perceive a change in a variable, it is expected that IS *detection* will be higher when using the tactile display (to augment the PM). However, the number of IS detections does not necessarily correspond to the number of IS identifications, as it is possible to detect an IS but to then identify it incorrectly. Consequently, it is equally important or potentially even more critical to measure IS identification, which corresponds to the percentage of IS detected and correctly identified. Most studies have achieved more than 90% accuracy for both metrics (see Multimedia Appendix 2 for more details). Therefore, the studies reviewed successfully demonstrated that conveying clinical information through tactile displays is possible. Nonetheless, the real significance of tactile displays for critical care can only be verified by conducting user testing with clinicians in real (or close to real) contexts of use. For example, the IS detection and IS identification of their tactile display were considerably lower in the study by Dosani et al [20] than in the study by Ng et al [19], although the same tactile display was used in both studies. The context of use in the study by Dosani et al [20] was in a pediatric unit with patients, whereas in in the study by Ng et al [19], the testing was conducted in a laboratory setting without patients.

**Figure 25:**
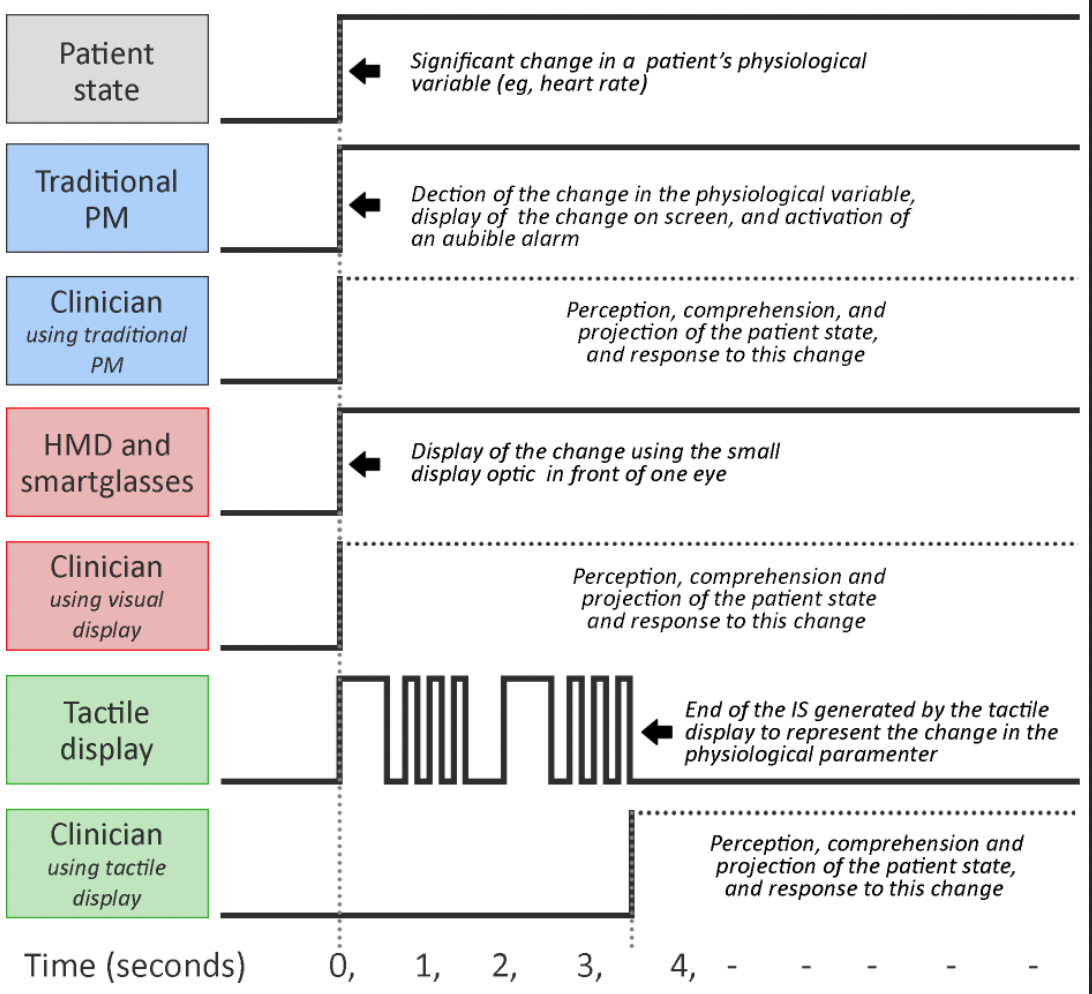
Timing diagram of patient state changes and clinician's response. With visual displays, the message is conveyed almost instantaneously. In contrast, in auditory and tactile displays, the message is conveyed through audio or vibration patterns, requiring more time to convey. HMD: head-mounted display; IS: interaction signal; PM: patient monitor.

*Response time* to a change in the patient state is one of the most common metrics used to assess clinicians’ performance with a new display, and this metric can be affected by several factors (eg, clinician’s experience, the tasks being performed in parallel with patient monitoring, and the monitoring device’s physical location in the room). Regarding response time, tactile displays alone have a clear disadvantage compared with visual displays, as the IS from a tactile display requires more time to be conveyed in its entirety to the clinician. For example, the duration of a tactile display IS can range from 0.5 seconds [22] to 3.5 seconds [17] (Figure 25). It is important to note, however, that tactile displays are intended to augment PMs in a critical care setting. Therefore, response time can be reduced by looking at the PM as soon as they feel the initial stimuli on their skin without waiting for the full IS to be conveyed.

### HMDs and Smart Glasses

#### Overview of the Studies

HMDs have also been considered for augmenting PMs in critical care. Our review identified 10 studies in which potential end users were asked to wear HMDs in simulated conditions or real practice. Most experiments were not able to provide robust evidence that HMDs or smart glasses led to an improvement in the user’s performance (eg, event detection, response time, and treatment efficiency) when used to monitor single patients during anesthesia or surgical settings [28,29,31]. However, promising results were achieved when HMDs were used to monitor multiple patients simultaneously [33,35].

#### Time Looking Toward the Patient

In all cases where the user’s gaze was monitored, it was verified that clinicians spent significantly less time looking toward the PM and more time looking toward the patient, while maintaining the same level of SA [29-31]. These findings indicate that HMDs can be useful from an ergonomics point of view in reducing the amount of clinician trunk and neck rotations associated with changing gaze, especially in environments where clinicians are physically constrained [29]. Beyond the possible comfort benefits of not averting their gaze from the patient, anesthesiologists could monitor changes in the patient’s skin pallor, chest movement, and other signs more quickly under these conditions. Therefore, HMDs may also enhance patient safety.

#### Usability and Ergonomics

Only Sanderson et al [28] and Liu et al [29] (experiment 1) asked participants about their preference in terms of PM used. These two studies presented conflicting results, with most participants in the study by Sanderson et al [28] preferring to use the HMD and most participants in the study by Liu et al [29] preferring not to use the HMD. However, it is important to note that participants in the study of Sanderson et al [28] were not monitoring a simulated patient but were supervising an actor who was monitoring a simulated patient, whereas in the study by Liu et al [29], participants were monitoring a simulated patient.

Regarding comfort and satisfaction, initial experiments with HMDs revealed a concern about the devices’ weight and wired nature, which affected the user experience negatively [28-30]. In general, this problem was not reported in studies involving smart glasses because of their lightweight form and their incorporation of wireless technology, except for 1 study [33]. Most participants in the experiments with smart glasses stated that they would like to use them in their work, and they would recommend their use to colleagues. This level of acceptance was mainly observed among younger participants [31,32,37]. However, some participants commented that wearing the HMD could distract them when they were doing tasks that required focus [33]. Others reported that they had to mentally focus on the data displayed by HMDs to observe and interpret it [29], which could generate eye fatigue. More research investigating the correlation between the use of these systems and eye strain or fatigue needs to be conducted to verify this finding.

### Smartwatches

Regarding the use of smartwatches for patient monitoring, McFarlan et al [38] have demonstrated promising results, which hopefully will lead to further studies investigating the feasibility and acceptance of these devices in the ICU. However, it is vital to keep in mind that the *bare below the elbows* policy, adopted in several hospitals in some jurisdictions, might impose an impediment in adopting these devices as they are currently designed. Researchers might have to identify ways of adjusting the design of these devices to be compliant with regulatory trends.

### General Comments on Wearable Devices for Critical Care

Most wearable devices (tactile displays, HMDs, or smartwatches) for critical care medicine (anesthesia, surgery, or the ICU) are intended to be used to augment current monitoring practices and not as a replacement. It is expected that, by adding another source of information, the likelihood of nurses and doctors missing a clinical event will be reduced, and they will be able to detect abnormalities faster. Researchers reported significant improvements in various metrics when participants used the PM plus a wearable display in comparison with participants using a PM only [17,28-33,35,38]. Some researchers explored the benefits of conveying information through multiple channels by developing multisensorial displays. These prototypes integrate, for example, auditory and tactile stimuli [27] or auditory, tactile, and visual stimuli [30] to inform the ICU nurse about patient alarms, thus increasing their SA and reducing alarm fatigue. Figures 3 and 4 illustrate how wearables can use different senses as communication channels. Beyond performance, conveying information through multiple channels might also be important for safety reasons if one of the wearable communication channels fails. Nonetheless, given their potential to overwhelming the users, the *suitability* of multisensorial wearable devices for critical care monitoring needs to be further investigated under conditions that reflect the proposed context of use.

It is important to note that enhancing the detection and identification of variable changes using wearable displays does not necessarily automatically translate into enhanced patient outcomes. Ultimately, clinical trials would be required to effectively demonstrate improved outcomes for patients.

### Limitations

Although all the studies reviewed presented wearable devices to augment patient monitoring in critical care, the studies diverged significantly in terms of the intended uses of the devices and the study designs adopted to evaluate them. Therefore, we acknowledge that, because of this heterogeneity in the literature, the ability to synthesize findings was reduced.

### Conclusions and Recommendations

This study aimed to review the literature on state-of-the-art wearable devices for critical care vital sign monitoring and to present the findings with a critical analysis of the usability and human factors performance of these devices. A total of 20 studies were identified: 9 on tactile displays, 9 on HMDs, 1 on a hybrid tactile and HMD display, and 1 on smartwatch displays. The studies on tactile displays have successfully demonstrated that these devices can be used to convey information on patient vital signs to critical care nurses and doctors. However, at this point, there is not enough evidence to indicate that tactile displays can positively impact the user’s performance compared with the PM only, and thus, more testing with critical care nurses and doctors is necessary. The issue of discomfort has been a significant challenge to be overcome in the design of these devices, with many participants reporting some level of discomfort when wearing tactile displays. Researchers should attempt to create more *finished* prototypes, ideally developed following an industrial design exercise, although this process can add significantly to the research cost.

The studies involving smart glasses for critical care patient monitoring have successfully demonstrated that these devices overcame the discomfort-related issues associated with their predecessor’s HMDs. When monitoring patients wearing HMDs or smart glasses, it was found that doctors spent more time looking at the patient and the surgical field than at the PM, compared with the case when they are using a PM only. This outcome can be potentially useful from an ergonomics point of view, in reducing the amount of trunk and neck rotations associated with changing gaze, especially in environments where clinicians are physically constrained. Additionally, this outcome can be useful from a patient safety point of view, in reducing the amount of time when the clinician is not directly observing the patient.

On the basis of our experience of reviewing these studies, we believe that future researchers can improve their investigations of novel wearable devices for critical care vital sign monitoring by (1) conducting experiments involving control (PM) and experimental displays, tested using the intended end users; (2) paying particular attention to comfort and technical performance aspects of their devices; and (3) using postexperiment interviews to enable the study to benefit from a qualitative analysis of issues such as comfort, user experience, and the likelihood of adopting the technology.

## Appendix

Multimedia Appendix 1 Terminology used in the review.

Multimedia Appendix 2 Summary of the experiments reviewed.

## Abbreviations

BP: blood pressure
CRS: comfort rating scale
HMD: head-mounted display
HR: heart rate
ICU: intensive care unit
IS: interaction signal
PM: patient monitor
PRISMA: Preferred Reporting Items for Systematic Reviews and Meta-analyses
SA: situation awareness
SUS: system usability scale
