# Supplementary material for: Augmenting Critical Care Patient Monitoring Using Wearable Technology: Review of Usability and Human Factors
Source: JMIR Hum Factors. 2021 May 25;8(2):e16491. doi: 10.2196/16491 (PMC8188324; doi:10.2196/16491)
Supplement: Multimedia Appendix 1 [file humanfactors_v8i2e16491_app1.docx]

### Terminology

The authors of the different studies reviewed in this paper used different terms to describe the same technology and different terms to describe the metrics used to evaluate the devices. For this reason, to reduce the potential confusion of the reader, we standardized the terminology used throughout the paper to facilitate a better understanding of the work described in this review. The list of these terms used is listed and described in the following section.

#### General Terms Used to Describe the Device or Study Design

- ALARM: Type of signal generated by the alarm system to indicate the presence (or occurrence) of an alarm condition (SOURCE: IEC 60601-1-8:2006).
- IS: Signal generated by the DISPLAY to convey information about the patient status to the operator (eg, a change in the VARIABLE value on the visual display, an audio ALARM, and a stimulus produced by a vibrotactile DISPLAY).
- DISPLAY: A device used to convey patient information (eg, VARIABLE IS or ALARM) to the clinician. Among the displays presented in this review, there are visual displays (eg, the information presented on a screen), auditory displays (eg, audio ALARM), and tactile displays (eg, the information presented through vibration or electrical stimulation).
- EVENT: Clinical situation that threatens patient safety such as infection, medication error, and heart attack. In some cases, the physiological abnormalities generated by the EVENT may not be detected instantly by the alarm system, in which case, the clinician may be able to identify the EVENT instinctively (eg, based on a change in skin pallor) or based on the patient information presented using a VARIABLE IS on the DISPLAY.
- HMD: DISPLAY worn on the head and delivering a variety of ISs (visual/auditory/tactile) to the user. For the purpose of this review, smart glasses (eg, Google Glass) will also be considered as a type of HMD.
- TACTOR: Small device present in tactile DISPLAYS that uses mechanical vibratory sequences to transmit patient status information to the clinician.
- VARIABLE: A vital sign VARIABLE (eg, HR, BP, and SpO_2_).

#### List of Outcome Measures Used by the Studies

- EVENT detection: The number of EVENTS detected by the clinician as a ratio of the total number of testing EVENTS.
- EVENT detection time: The time taken for the clinician to detect an EVENT.
- EVENT correction time: Time is taken to detect an EVENT, correctly identify it, take proper corrective actions, and ensure that all variable levels had been returned to within an "acceptable" range.
- Time looking toward the patient: Percentage of time clinicians spend looking at a specific location during the experiment (eg, time spent looking at the patient, time spent looking at the patient monitor, or time spent looking at other locations).
- Learnability: Amount of training necessary for the user to successfully interact with the DISPLAY. This can be measured by the time taken to finish the training session or the number of training sessions necessary to complete the training.
- IS detection: The number of ISs detected by the clinician as a ratio of the total number of ISs generated by the display (often expressed as a percentage). The number of ISs detected does not necessarily correspond to the number of ISs identified, as it is possible to detect an IS and identify it incorrectly.
- IS identification time: The time taken for the clinician to detect and correctly identify an IS. Measured from the moment the IS starts to the moment the clinician identifies it.
- IS identification: The number of ISs detected and correctly identified by the clinician as a ratio of the total number of ISs generated by the display (often expressed as a percentage).
- Response time: The time taken for the clinician to detect an ALARM or IS, correctly identify it, and take action in response. Measured from the moment the ALARM or CUE starts to the moment the clinician responds to it. In some particular cases, the *response time* was measured after the ALARM or IS sequence has finalized (eg, after a 3-seconds-long vibration IS had finished). In these cases, this information will be available to the reader.
- Task completion time: Time taken to complete a scenario from its beginning until all the required steps are completed.
- Treatment efficiency: Measurement of the clinician's success in treating a "patient." This the time the "patient’s” vital signs were at acceptable levels as a ratio of the total time the patient was monitored (often expressed as a percentage).
- Comfort: Clinician’s perceptions of the comfort of the DISPLAY. It is typically measured using a Likert-type scale (eg, scale ranging from 1=very uncomfortable to 7=very comfortable).
- Satisfaction: Clinician’s satisfaction with the DISPLAY. It is typically measured using a Likert-type scale (eg, scale ranging from 1=very dissatisfied to 7=very satisfied).
- Preference: Clinician’s opinions on which of multiple DISPLAY types they preferred (eg, in within-subjects, participants were asked which of the DISPLAYS tested they preferred the most).
- Usefulness: Clinician’s perceptions of how useful the DISPLAY would be if used in real life. Typically measured using a Likert-type scale (eg, scale ranging from 1=not useful to 7=very useful).
- Suitability: Clinician’s perceptions of the suitability of the DISPLAY. It is typically measured using a Likert-type scale (eg, scale ranging from 1=not suitable to 7=very suitable).
- Workload: Clinician’s perceived workload. It is measured using a NASA Task Load Index (NASA-TLX) scale.
- SA: Measurement of how situation-aware the clinician was during the experiment. Typically, a score is acquired using the Situation Awareness Global Assessment Technique in which the testing is paused randomly, and clinicians are asked questions about the VARIABLE values, patient state, etc.
