# Supplementary material for: Augmenting Critical Care Patient Monitoring Using Wearable Technology: Review of Usability and Human Factors
Source: JMIR Hum Factors. 2021 May 25;8(2):e16491. doi: 10.2196/16491 (PMC8188324; doi:10.2196/16491)
Supplement: Multimedia Appendix 2 [file humanfactors_v8i2e16491_app2.docx]

**Summary of the performance and usability experiments conducted using tactile displays.**

| Study | Display | IS^a^ detection | | IS identification | | Response time (seconds) | | Usability metrics |
| --- | --- | --- | --- | --- | --- | --- | --- | --- |
|  |  | mean | median | mean | median | mean | median |  |
| Ng et al [17]   - Displays: alarm HR^b^ anesthesia - Study: within-subject; N=10, no medical background | Auditory alarm | ^g^— | 92% | — | 96% | — | ^d^ 1.5 | ^e^ P=30% |
|  | Tactile forearm | — | ^f^↑ 100% | — | 98% | — | ^d^ 1.9 | P=40% |
|  | Tactile+auditory alarm | — | ↑ 100% | — | 97% | — | ^d^ 1.7 | P=30% |
| Ng et al [18]   - Displays: alarm anesthesia HR (no control condition) - Study: within-subject; N=26, no medical background | Tactile forearm | 95% | — | — | — | 5.69 | — | ^h^ C=5.4 |
|  | Tactile wrist | 94% | — | — | — | 5.43 | — | C=5.3 |
|  | Electro-tactile forearm | 89% | — | — | — | 5.41 | — | C=4 |
| Ng et al [19]   - Display: alarm anesthesia with 4 variables: NIBP_mean_^i^, MV_exp_^j^, PAP^k^, and EtCO_2_^l^ (no control condition) - Study: N=15 anesthesiologists with LW^m^ and HW^n^ | Tactile waist | ^m^ LW: 98%  ^n^ HW: 83% | — | LW: 97%  HW: 93% | — | LW: 9.1  HW: 9.5 | — | C=5.00  ^o^ S=5.07 |
| Barralon et al [21]   - Displays: alarm anesthesia with 6 variables: HR, NIBP^p^, SpO_2_^q^, AP^r^, EtCO_2_, and MV^s^ (no control condition) - Study: within-subject; N=28, no medical background | Tactile belt | — | — | 80% | — | 9.3  ^d^ 8.0 | — | — |
|  | Dorsal tactile | — | — | 81% | — | 10.0  ^d^ 5.6 | — | — |
| Ferris and Sarter [22]   - Displays: alarm and continuous anesthesia with 3 variables: MAP^t^, EtCO_2_, TV^u^ - Study: within-subject; N=16 anesthesiologists; 4 scenarios involving fluctuations of each monitored variable were used | Patient monitor | — | — | — | — | — | — | C=^v^NPI  S=NPI |
|  | Tactile alarm | — | — | — | — | — | — | — |
|  | Tactile continuous | — | — | — | — | — | — | — |
|  | Hybrid | — | — | — | — | — | — | — |
| Dosani et al [20]   - Display: alarm anesthesia with 4 variables: NIBP_mean_, MV_exp_, P_peak_^w^, and EtCO_2_ - Study: N=17 anesthesiologists | Tactile waist | 81% | — | 89% | — | — | — | S=4.8 |
| McLanders et al [25]   - Displays: continuous anesthesia with 2 variables: HR and SpO_2_ (no control condition) - Study: between-subjects; N=30, no medical background | Tactile upper arm; integrated | — | — | 95% | — | 1.1 | — | C=5.35 |
|  | Tactile upper arm; integrated | — | — | 93% | — | 1.5 | — | — |
| Cobus and Heuten [26]   - Display: alarm anesthesia, any abnormal variable (no control condition) - Study: within-subject; N=19 health care professionals | Tactile upper arm (using the chosen IS) | 100% | — | 100% | — | ^c^ 1.59 | — | NPI |
| Gomes et al [14] experiment 2   - Display: continuous anesthesia with 3 variables: SpO_2_, MAP, and EtCO_2_ (no control condition) - Study: within-subject; N=19 health care professionals | Tactile on the upper arm | — | — | LW: 98% HW: 96% | — | LW: 7.23 HW: 6.86 | — | — |
| Burdick et al [27]   - Display: multisensory anesthesia with 3 variables: HR, SpO_2_, and blood pressure - Study: within-subject; N=16, no medical background | Auditory display | NPI | — | NPI | — | NPI | — | — |
|  | Multisensory display | NPI | — | NPI | — | NPI | — | — |

^a^IS: interaction signal.

^b^HR: heart rate.

^d^Response time measured from the end of the interaction signal.

^e^P: preference (0%-100%).

^f^The arrows pointing up (↑) and down (↓) indicate that the metric tagged was superior (*P*<.05) when the intervention display was used compared with the traditional monitoring method. The ↑ symbol indicates increased values, whereas the ↓ symbol indicates reduced values. In studies where no traditional monitoring method (control) was tested, the statistical significance was not indicated.

^g^—: not measured or missing data.

^h^C: comfort scale (1=very negative and 7=very positive).

^i^NIBP_mean_: mean noninvasive arterial blood pressure.

^j^MV_exp_: expired minute ventilation.

^k^PAP: peak airway pressure.

^l^EtCO_2_: end-tidal carbon dioxide partial pressure.

^m^LW: low workload.

^n^HW: high workload.

^o^S: satisfaction

^p^NIBP: noninvasive arterial blood pressure.

^q^SpO_2_: oxygen saturation.

^r^AP: airway pressure.

^s^MV: minute ventilation.

^t^MAP: mean arterial blood pressure.

^u^TV: tidal lung volume.

^v^NPI: not possible to include because of incompatibility in the way the metric was evaluated compared with the other studies.

^w^P_peak_: peak airway pressure

Summary of the performance and usability experiments conducted using head-mounted displays or smart glasses.

| Study | Display | Event detection, mean (SD) | Event detection time (seconds), mean (SD) | Response time (seconds), mean (SD) | TLTP^a^, mean (SD) (%) | Usability metrics |
| --- | --- | --- | --- | --- | --- | --- |
| Sanderson et al [28]   - Displays: auditory display and HMD^b^ - Study: within-subjects, N=16 anesthesiologists | ^c^ PM | 50% (^d^ —) | 44 (21) | — | — | ^e^ P=6.2% |
|  | Auditory+PM | ^f^↑ 100% (—) | 33 (15) | — | — | P=25% |
|  | HMD+PM | 67% (—) | 40 (19) | — | — | P=31.2% |
|  | HMD+auditor+PM | ↑ 100% (—) | 28 (11) | — | — | P=37.5% |
| Liu et al [29], experiment 1   - Displays: HMD (near focus or far focus) - Study: within-subjects, N=12 anesthesiologists; left femoral-popliteal bypass, lower anterior resection, and left knee replacement scenarios were used | PM | 76% (3.7) | 72.8 (18.4) | — | 41% (2.3) | P=58% |
|  | HMD near focus+PM | 72% (3.7) | 77.6 (18.2) | — | ↑ 48% (1.7) | P=33% |
|  | HMD far focus+PM | 77% (5) | 78.3 (18.5) | — | ↑ 47% (2.0) | P=8.3% |
| Liu et al [29], experiment 2   - Display: HMD (self-adjusted focus) - Study: within-subjects, N=12 anesthesiologists; 4 scenarios were used: ischemia, excess sedation, light anesthesia, and hypovolemia (events 1, 2, 3, and 4, respectively) | PM | — | E1: 69.3 (18.6)  E2: 25.7 (6.8)  E3: 29.7 (5.0)  E4: 36.3 (8.5) | — | 93% (—) | — |
|  | HMD+PM | — | E1: 80.3 (17.5)  E2: 77.9 (11.8)  E3: 16.8 (3.3)  ↓ *P* =.034  E4: 13.9 (3.7)  ↓ *P* =.019 | — | ↑* 99% (—) | — |
| Liu et al [30]   - Display: HMD (self-adjusted focus) - Study: within-subjects, N=6 anesthesiologists; 4 weeks of monitoring real patients; 36 cases were collected (18 control and 18 experimental) | PM | — | — | — | 51% (—) | N/A^g^ |
|  | HMD+PM | — | — | — | ↑ 56% (—) | C^h^=4.9 |
| Liebert et al [31]   - Display: HMD (Google Glass) operating room - Study: within-subjects, N=14 surgical residents; 2 scenarios were used: thoracostomy tube placement (S1) and a bronchoscopy (S2). | PM | — | S1: 41.9 (19.5)  S2: 73.4 (65.3) | — | S1: 22% (12.1)  S2: 32% (19.2) | NPI^i^ |
|  | HMD+PM | — | S1: 31.8 (16.1)  S2: 64.6 (37.2) | — | ↓ ^j^ S1: 6% (6.8)  ↓* S2: 3% (8.3) | NPI |
| Iqbal et al [32]   - Display: HMD (Google Glass) operating room - Study: within-subjects, N=24 medical students (n=8 urology surgical trainees and n=5 consultant urologists); a laser prostatectomy simulation was used | PM | — | — | 51.5 (—) | — | NPI |
|  | HMD+PM | — | — | ↓* 35.5 (—) | — | NPI |
| Schlosser et al [33]   - Display: HMD (Vuzix M300) - Study: within-subjects, N=8 anesthesiologists monitoring multiple patients in a hospital | PMs | 7% (—) | — | — | — | NPI |
|  | HMD+PMs | ↑ 67% (—) | — | — | — | NPI |
| Cobus and Heuten [26]   - Displays: 3 alarm displays in ICU^k^ - Study: within-subjects, N=12 ICU nurses | Speakers | — | — | 3.71 (1.74) | — | C=4  S=4 |
|  | Peripheral lights (HMD) | — | — | 2.02 (0.6) | — | C=5.5  S^l^=5.5 |
|  | Auditory (HMD) | — | — | 3.37 (1.7) | — | C=5.5  S=5.5 |
|  | Tactile (HMD) | — | — | 3.15 (1.06) | — | C=4  S=4 |
| Klueber et al [34]   - Display: HMD (Vuzix M100) ICU - Study: between-subjects, N=57, no medical background | Auditory | N/A | N/A | — | N/A | — |
|  | Auditory+HMD | N/A | N/A | — | N/A | — |
|  | HMD | N/A | N/A | — | N/A | — |
| Pascale et al [35], experiment 1  Display: HMD alarm display (Google Glass) ICU.  Study: between-subjects, N=72, no medical background | Auditory alarms | N/A | N/A | 17.59 (3.95) | N/A | — |
|  | HMD+auditory alarms | N/A | N/A | ↓* 13.03 (1.87) | N/A | — |
| Pascale et al [35], experiment 2  Display: HMD (Vuzix M100) ICU.  Study: within-subjects, N=13 nursing students | Auditory alarms | N/A | N/A | — | — | — |
|  | HMD+auditory alarms | N/A | N/A | — | — | — |
|  | HMD+notifications | N/A | N/A | — | — | — |

^a^TLTP: time looking toward the patient.

^b^HMD: head-mounted display.

^c^PM: patient monitor.

^d^N/M: not measured or missing data.

^e^P: preference (0-100%).

^f^The arrows pointing up (↑) and down (↓) indicate that the metric tagged was superior (*P*<.05) when the intervention display was used compared with the traditional monitoring method. The ↑ symbol indicates increased values, whereas the ↓ symbol indicates reduced values. In studies where no traditional monitoring method (control) was tested, the statistical significance was not indicated. When the ↑ or the ↓ symbol comes accompanied by the (*) symbol, it indicates that the P value was lower than .005.

^g^N/A: not applicable.

^h^C: comfort scale (1=very negative and 7=very positive).

^i^NPI: not possible to include because of incompatibility in the way the metric was evaluated in comparison with the other studies.

^j^Time looking toward the patient monitor instead of the patient (%) values.

^k^ICU: intensive care unit.

^l^S: Satisfaction.
